# Patient-Specific Lattice Implants for Segmental Femoral and Tibial Reconstruction (Part 2): CT-Based Personalization, Design Workflows and Validation—A Review

**DOI:** 10.3390/biomimetics11020145

**Published:** 2026-02-13

**Authors:** Mansoureh Rezapourian, Anooshe Sadat Mirhakimi, Tatevik Minasyan, Mahan Nematollahi, Irina Hussainova

**Affiliations:** 1 Department of Mechanical and Industrial Engineering, Tallinn University of Technology, 19086 Tallinn, Estonia; tatevik.minasyan@taltech.ee (T.M.); irina.hussainova@taltech.ee (I.H.); 2 Department of Mechanical Engineering, McMaster University, Hamilton, ON L8S 4L7, Canada; 3 Department of Technical Physics, University of Eastern Finland, 70210 Kuopio, Finland; mahan.nematollahi@uef.fi

**Keywords:** patient-specific lattice implants, segmental bone defects, femur, tibia, computed tomography, additive manufacturing, porous scaffolds, finite element analysis, mechanical testing, in vivo validation, 3D-printed porous scaffolds

## Abstract

Patient-specific lattice implants (PSLIs) and modular porous scaffolds have emerged as promising solutions for treating diaphyseal segmental defects of the femur and tibia, particularly where conventional reconstruction methods fall short. This second part of our two-part review focuses on how current studies transform computed tomography (CT) and μCT datasets into architected lattice implants, as well as how these constructs are fabricated and numerically, mechanically, biologically, and clinically verified. We outline imaging pipelines, including Digital Imaging and Communications in Medicine (DICOM) acquisition, segmentation, contralateral mirroring, and Hounsfield Units (HU)–density–elasticity mapping, and show how these choices impact finite element (FE) models and print-ready geometries. Next, lattice design strategies and mixed-material concepts are compared and linked to specific additive manufacturing routes in metals, polymers, and bioceramics, such as laser powder bed fusion (LPBF), electron beam melting (EBM), fused deposition modeling (FDM), material jetting, and extrusion-based bioprinting. Methodological overviews of linear–elastic models and homogenized finite element (FE) models, along with bench-top mechanical tests, in vitro cell assays, in vivo animal studies, and early clinical series, are utilized to categorize the studies into four pathways: simulation (S), mechanical (E_mech), biological (E_bio), and validation (V). Based on the reviewed literature, we establish a general workflow for CT implants. We identify common gaps in the process, observe insufficient reporting of imaging and modeling details, note a lack of data on fatigue and remodeling, and recognize the limited size of clinical cohorts. Additionally, we provide practical recommendations for developing more standardized and scalable planning pipelines. Part 1 of this two-part review studied defect patterns, anatomical location, and fixation strategies for patient-specific lattice implants used in femoral and tibial segmental reconstruction, with emphasis on how defect morphology and subregional anatomy influence construct selection and mechanical behavior. It established a defect- and fixation-centered review that provides the clinical and anatomical context for the workflow and validation analysis presented in Part 2.

## 1. Introduction

Patient-specific, CT-planned lattice implants are rapidly transitioning from experimental prototypes to realistic options for the reconstruction of femoral and tibial segmental defects. However, the underlying engineering and validation workflows remain challenging and are only partially documented. While early reports demonstrated that CT-based virtual planning could guide the design of porous cages or lattice segments tailored to individual defects and fixation strategies, the associated pipelines, from image acquisition and segmentation through lattice generation, FE analysis, printing, post-processing, and bench or in vivo testing, differ widely between groups [1,2,3,4]. At the same time, the design space has expanded to include architected morphologies and resorbable scaffolds, all of which must satisfy simultaneous constraints on manufacturability, mechanical performance, and osseointegration [5,6,7,8,9,10].

FE simulations, alongside in vitro and in vivo experiments, have become essential for evaluating these constructs. However, they utilize varying material laws, boundary conditions, and outcome metrics, complicating comparison and translation [1,10,11,12,13]. Metallic lattices, along with polymer, ceramic, and composite and graded architectures are now produced by additive manufacturing (AM) techniques including laser powder bed fusion (LPBF, often reported as SLM (selective laser melting) in the cited studies), electron beam melting (EBM), fused deposition modeling (FDM), material jetting, and bioprinting, often followed by stress relief, hot isostatic pressing, or sintering; however, the relations between process parameters, microstructure, and construct-level performance are infrequently reviewed in a suitable manner [13,14,15,16,17]. These gaps slow the development of a reproducible CT–implant workflow that could be scaled beyond single-base case series.

Following the defect- and fixation-focused overview in Part 1 [18], this second paper concentrates on the design and validation pipeline. Together, Parts 1 and Part 2 form a two-part review series on patient-specific lattice implants for segmental femoral and tibial reconstruction. Part 1 described in detail how segmental femoral and tibial defects are classified by size and morphology, how fixation strategies (plates, nails, external fixation, Masquelet constructs, and megaprosthesis alternatives) are selected, and how these choices define the local mechanical environment. These aspects are therefore only briefly recalled here, and readers are referred to the companion Part 1 review for a full discussion. In this paper, we first summarize personalization and imaging workflows for long-bone reconstruction, including CT and μCT strategies, segmentation practices, and the definition of defect and host-bone regions of interest. We then review lattice architecture choices and the co-design of shells and interiors, as well as mixed-material and graded concepts, before studying materials and AM routes for metallic and non-metallic scaffolds. Finally, we compile and analyze methodological overviews from FE simulations, mechanical and biological testing, and animal or early clinical studies, and we conclude by outlining practical challenges and future directions for standardizing image-based lattice implant workflows.

## 2. Historical Evolution of CT-Planned Lattice Implants

### From Ilizarov to AM: The Evolving History of Femoral/Tibial Defect Repair

The modern treatment of segmental long-bone defects evolved from early internal plating to today’s patient-specific AM-ed scaffolds. Early research on bone plates by Hansmann, Lane, and Sherman evolved into standardized internal fixation through the Arbeitsgemeinschaft für Osteosynthesefragen (AO) movement, established in 1958, which formalized techniques, implant design, and surgeon education [19,20,21]. Load-bearing internal fixation was transformed in 1939 when Küntscher performed the first successful intramedullary (IM) nailing; IM constructs became the workhorse for diaphyseal femur/tibia stabilization [22,23]. In parallel, Ilizarov’s circular external fixation and bone transport methods (1950s–1960s) enabled the biological reconstruction of very large tibial/femoral defects, a strategy that is still validated in contemporary series [24,25]. Cross-sectional imaging then reshaped planning: the first clinical CT scan on 1 October 1971 (Ambrose/Hounsfield) culminated in precise 3D defect definition, templating and, decades later, patient-specific implant design [26,27].

Advancements in biologics and fixation have expanded reconstruction options: The induced-membrane (Masquelet) technique, developed in the mid-1980s, offers a reliable two-stage method for bone regeneration [28,29,30]. Locking-plate concepts matured in the early 2000s, enhancing fixation in osteoporotic and periarticular bone. The Reamer–Irrigator–Aspirator (RIA) system facilitated the harvest of large volumes of intramedullary autograft for segmental defects [31,32,33]. For infected/nonunion scenarios, antibiotic cement-coated nails emerged as an effective limb salvage option, while magnetic, motorized bone-transport nails (e.g., PRECICE BTN) reduced external-fixator time and expanded indications into oncology reconstructions [34,35,36,37]. Concurrently, surgeons trialed structural substitutes, such as cylindrical titanium mesh cages filled with graft (reported clinically in 2000), to bridge diaphyseal gaps [38,39]. Long-bone reconstruction is evolving from traditional solid hardware and grafts to advanced, patient-specific lattice architectures. These innovative designs are enabled by CT-based planning and modern AM.

Engineers now combine surface-lattice shells and interior scaffolds that incorporate screw/nail trajectories, graft windows, and functionally graded porosity to tune local stiffness and eliminate stress shielding. Different lattice families, including triply periodic minimal surfaces (TPMSs), strut-based, stochastic/Voronoi, hybrid, and multi-morphology architectures, are selected or blended to adjust anisotropy with femoral/tibial load paths while maintaining permeability [7,40,41]. Topology optimization and lattice libraries help shape geometries within the constraints of AM limits, such as minimum feature sizes and overhangs [42]. Meanwhile, fatigue verification and physiologically relevant FE loading, based on hip and muscle forces, are gradually integrated into the workflow, which follows the sequence starting from CAD, followed by FE analysis and 3D printing, then stress relief approach, and finally bench testing [12,43]. Recent studies demonstrate these principles in practice, optimizing lattice parameters and validating them through mechanical tests and in vitro/in vivo assays, supporting the move toward graded, load-sharing implants for long-bone reconstruction [12].

**Figure 1:**
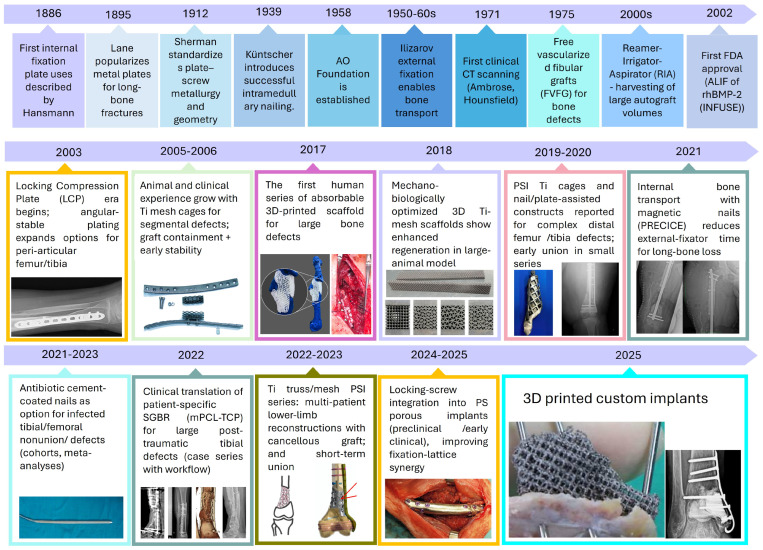
Historical evolution of reconstruction strategies for segmental femoral and tibial defects (1886–2025). The timeline from early internal fixation plates, intramedullary nailing, Ilizarov external fixation, and CT-based planning through to locking compression plates, titanium mesh cages, magnetic lengthening nails, and contemporary patient-specific solutions such as Ti lattice cages, mPCL-TCP SGBR scaffolds, and custom 3D-printed implants [3,51,52,53,54,55,56,57,58,59,60,61].

Over the last decade, CT-based CAD and AM have turned both metallic lattices and biodegradable scaffolds into realistic options for segmental femoral and tibial reconstruction [44,45]. Patient-specific porous titanium cages and lattice implants, often combined with Masquelet or plate/nail constructs and bioresorbable mPCL-TCP scaffoldillustrate how fixation, biology, and architected porosity can be integrated in a single reconstruction strategy. The clinical series based on these concepts are examined in detail in later sections here, completing the history from rigid fixation and biologic transport to imaging-based patient-specific lattices for load-sharing regeneration of femoral and tibial defects [2,46,47,48,49,50]. The historical progression from 1886 to 2025 is summarized in Figure 1.

## 3. Personalization and Imaging Workflow

**Figure 2:**
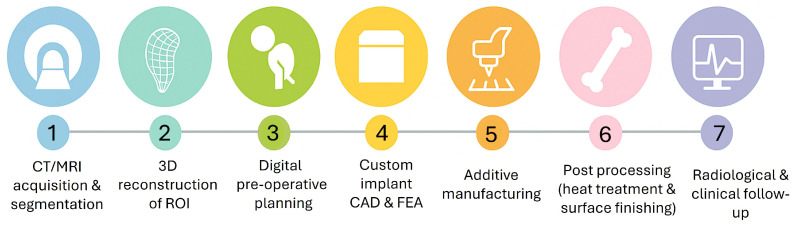
CT-to-implant workflow for patient-specific lattice implants in femoral and tibial reconstruction, starting from CT/MRI acquisition and segmentation, and ending with radiological and clinical follow-up.

Across the reviewed studies, CT–lattice personalization pipelines can be grouped into three main tracks. First, most clinical investigations use standard clinical CT of human femora and tibiae with millimetric slice spacing; these datasets feed both patient-specific implant designs and FE models [1,3,7,11,14,62,63,64]. Second, preclinical rat and sheep models utilize μCT for high-resolution imaging of morphology, allowing for longitudinal follow-up and quantification of both peri-implant and intraporous bone [4,6,9]. Third, several studies rely on reference or surrogate datasets such as composite femurs or Visible Human data to prototype workflows or validate mechanics when patient data are unavailable [15,65,66]. Figure 2 represents the CT-to-implant workflow for patient-specific lattice implants in femoral and tibial reconstruction, starting from CT/MRI acquisition and segmentation, 3D reconstruction of the region of interest (ROI), and digital preoperative planning, followed by custom implant CAD and FEA, AM, post-processing (heat treatment and surface finishing), and finally radiological and clinical follow-up.

More recent work illustrates a dedicated quality control branch, where μCT is used only to verify scaffold integrity rather than to build the patient model itself; for example, Lee et al. used a Scanco μCT 100 system (4.9 μm voxel size, 90 kVp, 200 μA, 140 ms) to assess the pore interconnectivity and strut fidelity of printed Ti lattices without compelling the CAD geometry [8]. Other authors have reported detailed clinical CT acquisition parameters like 2 mm inter-slice distance and 1,816 axial cuts before processing with Mimics 10.01, SolidWorks 2020, and ANSYS workbench 2021 [17] and have demonstrated open-source segmentation in InVesalius with fine voxels (0.115 × 0.115 × 0.600 mm) for ovine planning and follow-up quantification [10]. In complex reconstructions, Tetsworth et al. [2] showed that stage-1 Masquelet spacer CT, combined with contralateral mirroring, can be used to restore bone contours before sending DICOM data to the manufacturer for a patient-specific Ti cage. In the sheep model of Zhang et al. [4], μCT post-processing with HU thresholds between 1000 and 3885 HU and two regions of interest—a 2 mm peri-implant belt and an intraporous region—provided a standardized basis for quantifying bone ingrowth. Chang et al. [12] further demonstrated how lattice effective properties can be extracted via ANSYS Material Designer (Representative Volume Element (RVE) homogenization) from a CT-based distal femur model and then used to select a cuboctahedral lattice with 0.8 mm pillars at 45° that targets a bone-strain window of approximately 4000 μϵ at the interface.

Typical clinical pipelines report ∼512 × 512 image matrices with ∼1.0–1.5 mm slice thickness, followed by segmentation in Mimics, 3D Slicer, or Amira-Avizo; STL export with surface cleanup in Meshmixer, Geomagic, or Magics; and subsequent meshing and FE analysis in ANSYS or Abaqus [3,7,14,62,63,64,67]. Within this general pattern, several variations have been reported. Blázquez et al. used InVesalius to interactively process and organize ovine DICOM data, which has voxel sizes of 0.115 × 0.115 × 0.600 mm, before creating robocast HA (hydroxyapatite) scaffolds [10]. Vasanthanathan et al. analyzed dense CT metadata (2 mm slice spacing and 1816 cuts) prior to a Mimics–SolidWorks–ANSYS workflow [17]. Zhang et al. [4] employed Mimics Research 20.0 for segmentation and Abaqus 6.14 for FE analysis of a CT-based sheep to assemble femur, implant, and plate; while Lee et al. [8] used NX 12.0 as the primary FE environment, where clinical CT stood mainly as a verification endpoint, and μCT was reserved for QA of the printed scaffold. In a CT-based distal femur design study, Chang et al. [12] relied on Creo for CAD and ANSYS Material Designer to compute lattice elastic constants from RVEs before performing whole-bone FE analysis with 10-node tetrahedral elements under gait-like loading.

Generally, the introduction of defects into these models also follows two main strategies. In some studies, defects are taken directly from trauma or tumor CT, preserving the exact morphology and host bone condition [3,11]. In others, virtual osteotomies create standardized gaps—typically 50–90 mm diaphyseal defects—to enable controlled comparison of implant designs and fixation concepts [1,7,62,63]. Chang et al. [12] defined a 25 mm distal femur window located 55 mm from the joint line to parametrically study lattice and bone contact layers, whereas Blázquez et al. [10] modeled a 13 mm metatarsal segment removal in sheep and added coupler and graft-hole features directly in the 3D model to accommodate a robocast HA scaffold. Several clinical and preclinical workflows restore anatomy by mirroring the intact limb before designing patient-specific instrumentation or lattice implants [3,62]. In the Masquelet setting, Tetsworth et al. [2] used the cement spacer construct as the imaging target, mirrored the contralateral limb to re-establish native contours, and then generated a Ti cage that conforms both to the spacer envelope and to the mirrored bone geometry.

To capture bone mechanics more thoroughly, a subset of workflows applies HU–density-modulus mapping to the host femur or tibia, thereby reconstructing an inhomogeneous elastic field rather than assigning a single stiffness value to the entire bone [1,14,63,67]. Blázquez et al. [10] combined this approach with bone mineral density (BMD) calibration using QRM-BDC phantoms (0–0.8 g HA ${\mathrm{cm}}^{-3}$) and time-stamped CT follow-up scans, which allowed them to track bone formation and remodeling over time in an ovine model. On the implant side, Chang et al. [12] performed RVE-based homogenization in ANSYS Material Designer to derive effective elastic constants for the lattice, which were then embedded in a full-assembly FE model. In that work, the lattice topology and pillar dimensions were tuned to generate interface strains around 4000 μϵ, a target that is increasingly used in the first step of lattice designs to guide parameter choices such as pillar diameter and inclination [4,8,12].

Once the defect envelope has been defined, either from resected anatomy or from a mirrored reconstruction, lattice and PSI geometries are derived to occupy the available volume while respecting fixation paths and biological constraints. Designs range from stiffness-matched porous shells and graded unit-cell trabecular–mimetic architectures to surface lattices that incorporate countersunk screw channels and graft windows [11,14,62,64,67]. Pobloth et al. [68] reported honeycomb Ti-mesh constructs with soft and stiff variants optimized for strain transfer in a sheep segmental defect model and then translated similar Ti-mesh designs to clinical cases. Chang et al. [12] investigated cuboctahedral surface lattices whose pillar diameter and angle were tuned to stimulate favorable interface strain patterns, while Blázquez et al. [10] generated robocast HA scaffolds directly from an InVesalius-segmented ovine model. Geometries are typically exported as STL or STEP files. Manufacturing-ready parts are then prepared for layer-wise fabrication routes such as LPBF of Ti6Al4V, with minimum feature limits around 0.5 mm for walls or struts, and are often benchmarked against FE predictions using composite bones or digital image correlation before any translational step [1,14,15]. LPBF has been used not only for generic lattices but also for clinical Ti honeycomb meshes in both patients and sheep [68], and for Ti lattice constructs validated via biomechanical, in vitro, and animal tests [12]. Other manufacturing techniques include FDM-printed acrylonitrile butadiene styrene (ABS) prototypes derived from CT-Mimics-STL pipelines [69], printing from CT-based geometry with Cura slicing and ISO-style bench testing [17], and robocasting of 45 vol% HA scaffolds for ovine tissue engineering studies [10].

Clinical CT thus offers whole-bone context and realistic planning, but at the cost of coarser voxels; it benefits from contralateral mirroring and HU-based property mapping, although these introduce assumptions about bilateral symmetry and calibration sensitivity [1,3,14,63]. In contrast, μCT provides exquisite resolution of pore-scale features and bone ingrowth but is restricted to preclinical scales and limited fields of view [4,6,9]. QC-only μCT, even without direct patient-specific CAD, remains crucial for verifying pore interconnectivity and as-printed strut geometry in lattice implants [8]. Segmentation toolchains now explicitly report voxel dimensions and HU thresholds, as in the InVesalius-based workflows of Blázquez et al. [10], and standardized ROI definitions (e.g., 1000–3885 HU, 2 mm peri-implant belt, intraporous ROI) enhance the reproducibility of bone ingrowth metrics [4]. Choices of FE solver and meshing strategy (NX, ANSYS, or Abaqus; tetrahedral vs. hexahedral elements; bonded vs. contact interfaces), together with homogenization tools such as ANSYS Material Designer, materially affect predicted strain at the bone–lattice interface and, therefore, the inferred optimal topology [4,8,12]. Importantly, clinical applicability improves when authors document DICOM parameters (e.g., slice spacing and number of cuts), segmentation operations, HU–density mapping, export formats, and manufacturability constraints alongside bench-top or in vivo validation [1,4,11,14,15,17,62,63]. Table 1 summarizes, for each study, the imaging acquisition settings, segmentation and stacking tools, export formats, mirroring steps, and links to FE analysis and AM, distinguishing clinical CT from preclinical μCT and Quality Assurance (QA) scans. By unifying terminology and flagging unreported fields, this registry supports like-for-like comparison of personalization workflows and clarifies how CT data propagate into lattice design, mechanics, simulation, and manufacturing.

Despite the promise of CT-based personalization, we identified a critical lack of consistency in reporting across the 24 studies that we reviewed. To address this, we operationalized a Minimum Reporting Set (MRS) based on three pillars: Imaging Fidelity (A), Model Transparency (B), and Clinical Operability (C). While some pipelines provide high transparency in CT acquisition and HU-based material mapping, others omit essential segmentation and QA steps. Using this assessment framework, we classified current workflows into three readiness tiers, revealing that only nine studies provided sufficient documentation for full evaluation. A detailed description of the MRS scoring, tier definitions, and the itemized assessment of all included literature is provided in Supplementary Note S1 and Table S1.

## 4. Lattice Modeling and Architecture

**Figure 3:**
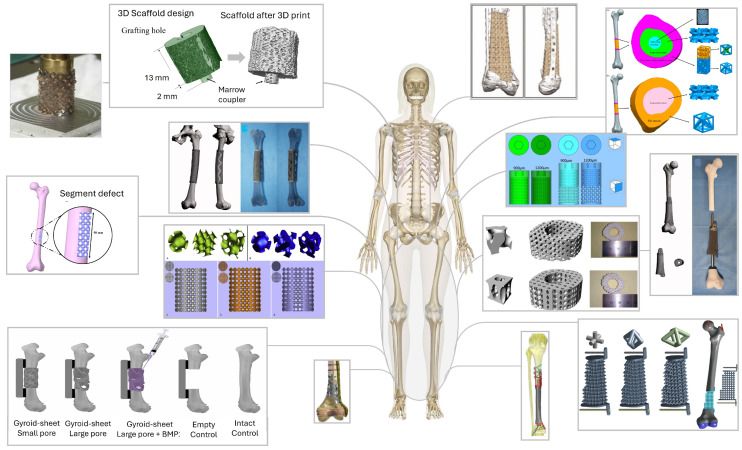
Overview of porous and lattice-based architectures used for segmental long-bone reconstruction. Examples include titanium lattice cages, mesh sleeves, TPMS and other architected scaffolds, and mPCL–TCP SGBR constructs applied around the femur and tibia, illustrating the diversity of porous architectures and anatomical locations addressed in current clinical and preclinical work [2,3,5,6,7,10,13,62,63,66,71,72,73].

As summarized in Table 2, lattice regions in femoral and tibial scaffolds are typically defined through a small set of geometric design variables rather than through complex generative rules. Most studies adopt relatively simple periodic or honeycomb frameworks with cylindrical scaffolds or struts and unit-cell sizes in the sub-millimeter to few-millimeter range, adjusting only the strut diameter, pore size, and overall porosity to tune stiffness and strength [1,8,12,14,15,63,67,69,70]. In the included femur/tibia dataset, TPMS- and shell-based lattices are reported less frequently; this distribution reflects the selection scope of this review rather than the broader TPMS literature [5,6,9,11,16,62,64]. Several reports emphasize highly porous cages or mesh constructs to contain large volumes of graft within a stable Ti envelope, rather than to achieve a finely graded stiffness profile [2,3,10,16,68]. The range of lattice and scaffold architectures currently explored for segmental femoral and tibial reconstruction is illustrated in Figure 3.

Across these studies, pore sizes generally fall in the few-hundred to roughly 1500 μm range, with designed porosities or relative densities (RDs) spanning roughly 40–90% when reported [1,6,8,10,13,67,69,70]. In many cases, the lattice is intentionally kept uniform along the defect segment, and mechanical tuning is achieved by switching between a small number of discrete soft and stiff scaffolds, rather than by introducing continuous spatial grading [1,4,12,13,63,68,69,70]. True functionally graded implementations, where porosity, morphology, or material varies between bearing zones and interface regions or follows CT-based stiffness maps, are still relatively rare, relying on prescribed radial or axial profiles or on segmentation of the implant into modules with different architectures [14,16,62,66,67]. Design objectives are dominated by mechanical criteria (matching or approximating intact bone stiffness, limiting von Mises stress below the alloy yield, controlling interfacial micromotion), while permeability, specific surface area, and detailed pore-level targets are only occasionally quantified, for example, via surface area-to-volume ratio (SA/VR) metrics or mechanobiological simulations of strain energy distribution [10,15]. The CAD and FE toolchain is correspondingly pragmatic, typically combining medical image segmentation (e.g., Mimics, ScanIP, InVesalius) with general-purpose CAD packages (SolidWorks, Creo, Fusion∼360, CATIA) or FE environments (Abaqus/CAE, ANSYS), and in several case reports the commercial lattice generator is not specified at all [1,2,3,8,9,10,11,12,15,16,62,63,67,68,69]. Table 2 compiles, for each study [n], the type of lattice used, key geometrical inputs (unit-cell size, strut thickness, pore size, relative density/porosity, surface area-to-volume ratio), and any applied gradients together with their drivers. It also records the stated design objectives and CAD environments, allowing direct comparison of how different groups tuned lattice architectures to meet mechanical and biological targets in long-bone defect reconstruction.

Because stiffness is reported heterogeneously (effective modulus vs. construct stiffness vs. hardness proxies), and often under different loading modes and boundary conditions, we avoid deriving a single consensus stiffness target and instead interpret stiffness indirectly via the mechanobiological strain/micromotion anchors summarized in Part 1 Table 1. In this review, lattice architectures are grouped into three modeling families: strut/beam lattices defined by explicit CAD primitives, TPMS (sheet/skeletal) lattices defined by implicit surfaces, and hybrid concepts (e.g., solid shells combined with porous infill or region-wise mixed morphologies). TPMS lattices are commonly generated using a level-set (implicit) formulation, where the lattice surface is obtained as an iso-surface of a periodic function f(x,y,z) = 0), and thickness is applied by offsetting the iso-surface to form sheet or skeletal variants. This classification is used to interpret how the included patient-specific workflows for femur/tibia reporting and lattice modeling [74] are implemented.

### Materials and Manufacturing

To provide a comprehensive overview of the AM methods of biomaterial printing, a summary table (Table 3) was prepared. The table highlights the initial materials, manufacturing processes, devices, primary process parameters, post-processing stages (if applied), the final shape of the 3D-printed parts, the bone segment they were intended to replace, and the characterization techniques used for the manufactured implants. This overview reveals the main trends in material choice, manufacturing methods, and post-processing across the listed studies. The biomechanical and biological characterization data were presented in previous sections and, hence, are not presented here. Research papers primarily focused on computational analysis were excluded from this table.

On the metallic side, most long-bone implants are produced from titanium-based powders by powder bed fusion. Ti6Al4V (often in medical extra-low interstitial (ELI) grades) is the default alloy for highly porous cages, meshes, and lattice segments manufactured by SLM/LPBF, direct metal laser sintering (DMLS), or EBM, as in the work of Wieding, Yavari, Pobloth, Tetsworth, Wong, Rana, Kelly, Zhang, Wu, Lee, and Chang [1,2,3,4,6,8,11,12,14,68,70]. More recently, pure Ti Grade∼ II and β-type Ti19Nb14Zr have also been used where specific stiffness or modulus targets are required [8,13,16]. These components range from open-porous test coupons and generic cylinders to fully patient-specific cages and interlocking block systems designed for segmental femoral or tibial reconstruction [2,3,8,12,14,16]. Typical process descriptions emphasize layer thickness, laser power, scan speed, and hatch spacing when available, but several clinically oriented reports simply specify the commercial machine and quality system rather than full parameter sets [2,3,11,68].

Alongside these metal implants, a broad set of polymeric, ceramic, and composite systems are used, where the emphasis is on regenerative scaffolds, architectural prototypes, or physical femur models. Photopolymer-based lattices and calibration specimens are fabricated by PolyJet material jetting or desktop SLA from VeroWhitePlus and UV-curable resins, including HA/CPP-filled formulations intended for TPMS and graded scaffolds [15,64]. Extrusion-based printing is used for both bioceramic and polymer architectures: polylactic acid (PLA)–β-TCP–HA composite slurries and HA inks are dispensed to create degradable scaffolds with spherical, gyroid, or strut-based architectures [5,10], while ABS, CF-PEEK, and PCL filaments are built into femur surrogates and modular scaffold “bricks” by FDM-type printers [17,66,69]. An indirect route was illustrated by Charbonnier et al., who first printed sacrificial wax molds using inkjet Drop-on-Demand (DoD) and subsequently infiltrated them with HA slurry before debinding and sintering [9]. In all of these cases, the printed architecture is closely tied to its intended role: load transfer and fixation for Ti-based cages, or space-making and osteoconductivity for HA-rich and polymer-based structures.

Post-processing steps follow directly from the chosen material class and process. For powder-bed-fused Ti alloys, the dominant steps are stress relief or annealing heat treatments, hot isostatic pressing where specified, and secondary operations such as EDM, abrasive blasting, and chemical etching to remove supports, relax residual stresses, and tune surface roughness [3,4,6,8,12,14,16,45,70]. Ceramic and composite scaffolds printed from slurries or inks undergo drying, debinding, and high-temperature sintering cycles to achieve densification and phase stability [5,9,10], while FDM parts typically require only support removal and, in some cases, assembly into defect-spanning constructs [17,66,69]. Cleaning and sterilization protocols are reported intermittently, mostly in in vivo or translational studies [10,13].

The characterization strategies listed in Table 3 focus on verifying that the manufactured construct matches its design intent in terms of architecture, composition, and basic properties. Image-based tools such as scanning electron microscopy (SEM), backscattered-electron scanning electron microscopy (BSE-SEM), micrographs analyzed in ImageJ, digital image correlation, and imbibition tests are used to quantify pore morphology, wall thickness, surface topography, and fluid uptake [6,8,9,10,15,68]. X-ray diffraction (XRD) and Fourier-transform infrared spectroscopy (FTIR) provide phase and chemical information for HA-containing systems [5,9]. At the same time, density and relative density are established through unit-cell calculations, displacement methods, or He pycnometry [13,64,69]. For metal lattices, nanoindentation and coupon-level testing are occasionally used to link thermal histories and post-processing to local stiffness and hardness [16]. Together, these entries show how material choice, additive route, and characterization protocol are combined into complete manufacturing workflows that underpin the mechanical and biological outcomes discussed in the rest of this paper.

One major practical limitation in AM-fabricated porous metallic implants, especially for LPBF lattices with small unit cells, is that the as-built geometry often deviates from the CAD model, and these deviations can measurably shift stiffness, strength, and local stress hotspots. Among the reviewed studies, only a few explicitly quantify this. Charbonnier et al. [9] quantified manufacturing fidelity by comparing μCT reconstructions to the CAD model (using a dimensional matching workflow), showing that deviations can be in the order of tens of microns and are not spatially uniform; they also emphasized that strut/beam collapse depends on orientation and defect presence, and that collapsed beams reduce structural stiffness. In a load-bearing gyroid implant workflow, Kelly et al. [6] similarly compared printed topology against CAD using μCT and noted that even when the topology is very similar, small-feature lattices exhibit systematic mismatch mechanisms; partially adhered particles and residual powder reduce the effective porosity (reductions of up to ∼10% are commonly reported), and this effect becomes stronger as pore size decreases due to increased surface area for particle adhesion, meaning that the as-built porosity can be lower than the nominal CAD porosity.

From the modeling perspective, Wieding et al. [75] explicitly acknowledged that idealized CAD may misrepresent real manufactured geometry; therefore, they adapted the scaffold geometry based on microscopy observations before FE analysis. Yavari et al. [70] reported a nominal pore size but determined the actual microarchitecture dimensions using micro-CT, highlighting that dimensional metrology is required rather than assuming the CAD dimensions. In contrast, other implant studies [4,8] frequently use micro-CT primarily for outcome assessment (e.g., bone/implant evaluation) without clearly reporting CAD-to-built deviation metrics, leaving a gap in how manufacturing fidelity is linked to mechanical performance. Overall, these studies support a clear reporting recommendation for biomedical porous metals; whenever mechanical performance is claimed—especially for small-unit-cell lattices—authors should report as-built verification (μCT/XCT), quantitative deviation metrics (e.g., strut thickness/pore size shifts), and dominant defect modes, because these factors can explain discrepancies between idealized FE predictions and experimental outcomes [7].

## 5. Methodological Overviews: Simulation, Experimental, and Clinical Studies

### 5.1. Numerical Simulation Frameworks for Lattice-Based Segmental Reconstructions

The simulation studies summarized in Table 4, when read alongside the anatomical overview, reveal a range of numerical frameworks that support lattice-based reconstruction of femoral and tibial defects. At one end are models that treat the scaffold or unit cell as the main mechanical object of interest, often using simplified boundary conditions such as uniaxial compression between rigid plates to extract effective stiffness or stress–strain responses [5,8,15]. At the other end are fully assembled bone–implant constructs in which plates, nails, screws, and porous regions are embedded into CT-based femora or tibiae and loaded under single-leg stance or three-point bending configurations [1,4,11,12,63,65]. Homogenized or orthotropic representations of the lattice are options that balance detail and efficiency. They offer a simpler way to model the structure while still reflecting its overall response [12,15].

Methodologically, the frameworks differ most clearly in three ingredients: material laws, boundary/loading conditions, and validation strategy. Most studies adopt linear elastic, isotropic properties for bone and metallic components, sometimes enriched by CT-based density–elasticity mapping or regionally graded elastic moduli within the scaffold to emulate stiffness gradients [14,16,62]. Loading ranges from idealized axial compression or three-point bending to more physiological single-leg stance setups with joint reaction and muscle equivalent forces applied at the hip or knee, and constraints imposed at the distal femur or tibia [1,11,12,65,68]. Contact definitions span perfectly bonded interfaces, “no-separation” contact, and frictional formulations, reflecting different assumptions about micromotion and osseointegration. Validation ranges from purely in silico parameter sweeps, through calibration against uniaxial scaffold tests, to full construct-level comparisons with strain gauges, digital image correlation, or large-animal models [4,12,15,17,76].

**Figure 4:**
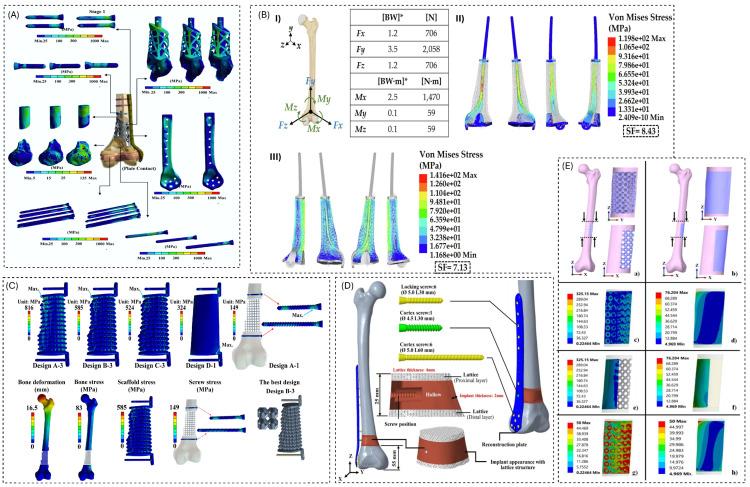
Representative FE results for segmental long-bone reconstruction: (**A**) Presentation of peak stresses at the lattice–stem junction, bone–implant interface, and around screws [3]. (**B**) Comparison of lattice and solid implant segments and stress and deformation distributions under compressive loading and bodyweight (BW); (**I**) Schematic of peak multi-axis internal forces and moments acting on the femur during walking for a 60-kg patient. (**II**) Finite element analysis of stress distribution under combined three-axis loading with applied safety factors in the stem and (**III**) in the lattice region [77]. (**C**) Comparison of bone deformation, bone stress, scaffold stress, and screw stress across multiple designs [63]. (**D**) Diagram of the distal femur reconstruction model; the hollow implant with proximal and distal lattice layers, fixation plate, and screw configuration [12]. (**E**) Assembly of the critical-size bone defect with solid and porous scaffolds on femur bone to demonstrate the stress transfer patterns (**a**) Porous scaffold implanted in the bone, (**b**) Solid scaffold implanted in the bone, (**c**–**h**) FE results of stress distribution in both solid and porous scaffold under physiological load conditions [7].

Within the design–validation workflow focused on in this paper, these simulation frameworks serve distinct roles. Scaffold- or unit-cell-level models enable rapid exploration of porosity, architecture, and gradient strategies before committing to patient-specific geometries. Construct-level FEA of femur or tibia segments guides decisions on plate vs. nail fixation, lattice stiffness targets, and acceptable ranges of bone strain, as well as helping to identify regions at risk of stress shielding or hardware overloading [7,11,16,63,68]. Finally, models that are explicitly coupled with mechanical tests or in vivo data provide the first elements of a verification chain, but they still rarely address fatigue life, time-dependent remodeling, or uncertainty in boundary conditions and material parameters. The remainder of this section, therefore, uses the classification in Table 4 to discuss how current simulation practice can be integrated more systematically with experimental mechanics and clinical follow-up in future patient-specific workflows. These distinctions are used in the subsequent simulation-focused subsection to compare modeling assumptions, identify recurring simplifications, and highlight gaps such as limited treatment of muscle forces, time-dependent remodeling, or uncertainty quantification. Typical FE pipelines for designing and evaluating lattice-based reconstructions of femoral and tibial segmental defects are illustrated in Figure 4.

Across the reviewed simulation studies, the suitability of material models, boundary conditions, interface treatments, and solver choices depends strongly on the model’s intended decision, because fidelity varies widely across these dimensions. Several studies adopt linear elastic, isotropic bone and implant properties with simplified interfaces (often bonded or tied), which are computationally efficient and well suited for screening large design spaces and ranking relative stress or stiffness trends but cannot directly support claims related to strut failure, crack initiation, fatigue degradation, or interface loosening [1,67]. Other works increase clinical representativeness by incorporating patient-specific CT-based geometry, staged-healing representations, and more physiological loading, such as single-leg-stance-type joint reactions and muscle forces [78] or CT-derived density-to-modulus mapping combined with hip reaction forces to study load sharing and stress shielding [63].

Lattice representation further defines use cases: explicit strut-level geometry preserves local stress hotspots but becomes computationally prohibitive at the construct level, so several studies employ homogenized or orthotropic equivalents when modeling whole bone–implant fixation systems. Importantly, Entezari et al. [15] combined orthotropic homogenization with quantitative experimental benchmarking (including digital image correlation) and frictional contact models, thereby increasing credibility when interface mechanics and micromotion are central outputs. In contrast, many construct-level models simplify screw and plate fixation under bonded or no-separation conditions, which is appropriate for assessing global load transfer and stiffness trends but tends to underestimate relative motion and the risk of loosening at interfaces [63,78].

Boundary conditions similarly range from test-mimicking setups (e.g., three-point bending used to enable direct FE–experiment stiffness comparison) to more physiological or standards-based loading, such as ISO 7206-4 compression or joint-reaction-based loading to evaluate stress shielding and osseointegration risk [4,12,17]. Solver selection aligns with these goals, with ANSYS and Abaqus commonly used for static or nonlinear contact analyses, while cyclic loading is rarely treated as fatigue-life prediction and is instead limited to short stability checks, where reported [16]. Finally, validation practices are uneven: some studies quantitatively anchor simulations to experiments (e.g., compression tests, strain gauges, or DIC) and report agreement trends [12,15,16], whereas others provide limited direct FE-to-data comparison [67,78]. As a result, most simulation outcomes are most defensible for comparative design ranking under stated loading assumptions, while they are less defensible for predicting long-term durability or failure unless nonlinearity, cyclic loading, interface evolution, and experimental validation are explicitly addressed.

While FE modeling is widely used to assess the mechanical performance of personalized implants and scaffolds, the underlying assumptions and modeling fidelity vary substantially across studies. To explicitly compare and contextualize these limitations, we evaluated each FE-based study using a small set of credibility indicators, including analysis type (static or quasi-static), interface modeling (bonded, no-separation, or frictional), bone representation (homogeneous vs. CT-based heterogeneous), inclusion of geometric or material nonlinearities, and the presence of experimental or benchmark validation. The results of this assessment are summarized in Supplementary Table S2, which ranks FE studies into three fidelity tiers: Tier 1 studies typically rely on linear elastic material laws, static loading, and simplified or undocumented interfaces, lacking quantitative validation. Tier 2 studies report key improvements such as CT-based material mapping, explicit contact definitions, or mesh convergence checks but remain limited by static loading or incomplete validation. Tier 3 studies combine clinically representative constructs with quantitative experimental validation and, in some cases, different bone modeling or advanced interface treatment. This structured comparison makes explicit how common simplifications—such as linear elasticity, static loading, and idealized interfaces—affect the interpretability and translational strength of FE predictions, rather than treating all numerical studies as methodologically equivalent.

### 5.2. Experimental Overview: Mechanical Tests, Biology, and In Vivo Validation (E_mech, E_bio, V)

**Figure 5:**
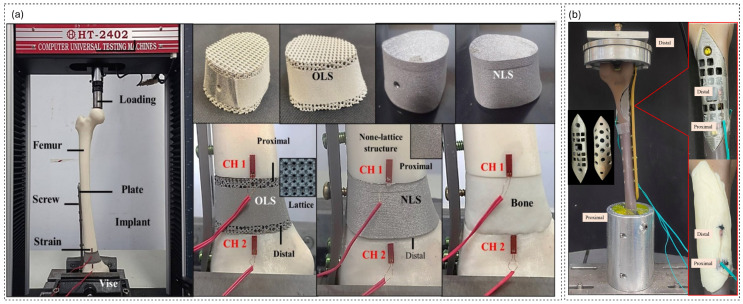
Experimental setups for mechanical testing of lattice-assisted reconstructions: (**a**) Biomechanical testing comparing OLS (outer-lattice structure) and NLS (non-lattice structure) implants. The femur is loaded axially to evaluate how lattice and non-lattice implant designs influence load transfer [12]. (**b**) Experimental setup of the implant–bone construct under cyclic axial compressive loading, with close-up views of the proximal and distal implant regions [11].

Table 5 assembles the experimental layer of the workflow, from simple coupon tests to animal models and early clinical evidence. At the most fundamental level, a series of studies report E_mech data on isolated scaffolds or surrogate bones: porous Ti specimens for FE calibration [1,14,16], polymer or composite lattices tested in compression before and after degradation [5,69], and CT-derived CF–PEEK or ABS femur substitutes designed to reproduce whole-bone stiffness for plate-testing protocols [17,69]. These experiments supply effective moduli, strength, and energy-absorption values, but they also reveal how process parameters and microarchitecture influence stiffness and collapse behavior, which can then be fed back into numerical models or design rules. Figure 5 also represents a samples of experimental setups for mechanical testing of lattice-assisted reconstructions.

A second group combines mechanical testing with biological readouts or explicit model–experiment comparisons. In this category, porous Ti and ceramic implants are implanted in rats, sheep, or pigs and assessed using a combination of ex vivo torsion or bending testing, μCT, histology, and gene expression [4,6,9,12]. Lattice-based Ti cages and scaffolds are shown to restore a substantial fraction of native stiffness while supporting bone ingrowth and, in some cases, near-complete bone–implant interface fusion over follow-up periods of 8–24 weeks [4,6,9]. Other works focus on validation (V) in a stricter sense: homogenized or RVE-based material models are checked against DIC fields or strain-gauge data on femur–implant constructs [10,11,12,15], and in vivo force and gait measurements are used to quantify how bioceramic or Ti lattices share load with regenerated bone during healing [10,13]. Together, the studies in Table 5 illustrate how mechanical characterization, biological assays, and in vivo monitoring can be integrated into a stepwise validation chain, but they also underline current limitations: small cohort sizes, relatively short follow-up times, and only a handful of examples where E_mech, E_bio, and V are combined within a single, fully documented workflow.

### 5.3. Translational and Clinical Overviews (C)

**Figure 6:**
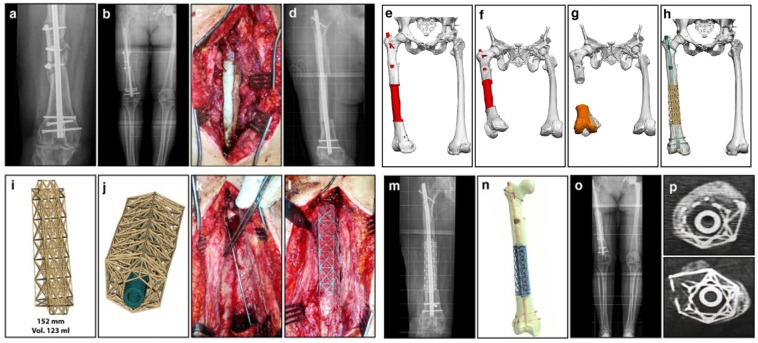
Example of clinical application of a patient-specific lattice cage for a diaphyseal femoral segmental defect; (**a**–**d**) Preoperative radiographs show the defect and previous fixation. (**e**–**j**) CT-based segmentation and virtual planning of the resection and custom lattice cage around an intramedullary nail. (**k**,**l**) Intraoperative images illustrate implantation of the cage and graft. (**m**–**p**) Postoperative radiographs and CT confirming construct position, bone graft, and defect filling [79].

Finally, Table 6 marks studies that report genuine clinical translation, from individual case reports to small series or feasibility trials. Tetsworth et al. [2] used patient-specific 3D-printed Ti cages within a Masquelet protocol to reconstruct massive post-traumatic femoral segmental defects, achieving limb salvage and union in all reported cases, while Zhang et al. [4] applied individualized porous Ti implant–bone fusion constructs to multi-centimeter defects of the femur, pelvis, and spine, with encouraging early fusion and implant survival. Although this clinical case study remains small compared to the number of purely preclinical or numerical studies, it is crucial for linking the review in real indications (trauma, tumor, infection), defect sizes and locations, reconstructive strategies (cages, lattice implants, modular blocks, resorbable scaffolds with vascularized tissue transfer), and patient-based designs such as union, reoperation, and complications. The clinical subsection therefore uses the C-flagged studies to outline current indications, typical success and failure modes, and the extent to which advanced lattice and TPMS designs have moved from conceptual prototypes towards routine use in segmental bone defect reconstruction. A representative clinical workflow for patient-specific lattice cage reconstruction of a femoral segmental defect is shown in Figure 6.

## 6. Critical Limitations and Current Gaps

Despite the rapid growth of CT-based lattice implant design for femoral and tibial segmental defects, a critical analysis of the literature reveals several recurring limitations that constrain interpretation, reproducibility, and clinical translation. These limitations arise at multiple stages of the workflow, including image-based personalization, finite element (FE) modeling, additive manufacturing (AM), validation strategies, and clinical deployment. Rather than being isolated shortcomings of individual studies, many of these issues appear systematically across the field and, therefore, warrant explicit synthesis.

### 6.1. CT and Personalization Limits

CT-based personalization is frequently presented as a defining strength of patient-specific lattice implants; however, its implementation is often incomplete or inconsistently reported. While many studies rely on CT-derived geometry to define defect shape and implant fit, only a subset of them extend CT information beyond geometry to inform material heterogeneity through HU–density modulus mapping. As a result, most FE models treat bone as homogeneous cortical and cancellous regions, even when patient-specific CT data are available. This simplification limits the ability to capture local stiffness variations that govern load sharing, stress shielding, and interface strain.

Segmentation and model generation steps are another source of uncertainty. Threshold selection, smoothing, hole filling, and surface defeaturing are rarely reported in sufficient detail to enable reproducibility, and the sensitivity of results to segmentation choices is almost never explored. In addition, most workflows assume static anatomy, neglecting postoperative remodeling, resorption, or changes in defect morphology over time. Consequently, CT-based personalization is typically limited to geometric conformity at implantation, rather than representing a time-dependent, mechanically evolving biological system.

### 6.2. FE Modeling and Validation Limits

Finite element modeling is widely used to compare lattice architectures, fixation strategies, and material choices, but model fidelity varies substantially. Many studies employ linear elastic material laws, static or quasi-static loading, and simplified interface conditions (e.g., bonded or no-separation contacts), which are computationally efficient and suitable for relative design screening. However, such assumptions inherently limit conclusions related to crack initiation, strut failure, interface loosening, or long-term durability.

Validation practices are similarly uneven. While some studies quantitatively benchmark FE predictions against mechanical tests (e.g., compression, bending, strain-gauge measurements, or digital image correlation), a large fraction rely only on qualitative agreement or provide no direct validation at all. Fatigue damage accumulation, cyclic degradation, and time-dependent failure modes are almost entirely absent from FE models, even though several authors have acknowledged their critical importance for clinical relevance. As a result, most simulations are best interpreted as comparative tools for ranking designs under idealized loading conditions, rather than as predictive models of long-term implant performance.

### 6.3. Additive Manufacturing and As-Built Deviations

One major but overlooked limitation in the reviewed literature is the discrepancy between as-designed lattice geometries and as-built structures produced by powder bed fusion. Manufacturing-induced deviations such as strut thickening, pore closure, partially fused powder particles, surface roughness, and internal defects—are particularly pronounced in small-unit-cell lattices and can significantly alter stiffness, strength, and fatigue behavior.

Only a limited number of studies explicitly quantify these deviations using μCT or SEM-based reconstruction and compare them against the original CAD models. Where such analyses are performed, they demonstrate that local geometric errors are non-uniform and orientation-dependent, and that even small deviations can reduce effective porosity or shift stress concentrations. The majority of FE studies nevertheless assume idealized CAD geometry, which may partially explain discrepancies between simulated and experimental results. Without routine as-built verification and geometry-informed modeling, the mechanical predictions of lattice implants remain optimistic and potentially non-conservative.

### 6.4. Clinical Translation Constraints

Beyond technical modeling challenges, several barriers impede clinical translation. Most studies focus on single-patient or single-defect scenarios, limiting generalizability. Surgical constraints, intraoperative variability, and revision scenarios are rarely considered explicitly in either design or simulation. Infection risk, aseptic loosening, and failure under off-axis or accidental loading are acknowledged conceptually but seldom incorporated into modeling or testing frameworks.

In addition, regulatory and workflow constraints—such as data handoff between imaging, design, manufacturing, and clinical teams—are rarely discussed in detail. Validation is often limited to bench-top mechanical tests, with relatively few studies providing longitudinal in vivo or clinical follow-up. Together, these gaps highlight a disconnect between computationally optimized designs and the practical requirements of robust, scalable clinical deployment.

## 7. Challenges and Future Directions

Despite the rapid progress from Ilizarov frames and bulk metal segments to CT-planned, AM-ed lattice implants, several fundamental challenges remain before patient-specific lattice implants (PSLIs) can become routine in femoral and tibial reconstruction.

### Clinical Workflow and Translational Barriers

A first major challenge is integrating CT-based planning and lattice design into real clinical timelines. Most studies in this review assume idealized conditions: clean CT datasets, generous design time, and close collaboration between engineers and surgeons. In practice, trauma cases, oncological resections, and revision surgeries often require decisions within days rather than weeks. Robust, semi-automatic pipelines for segmentation, defect classification, implant design, and validation must be integrated into clinically acceptable timelines and hospital information systems. Furthermore, regulatory pathways for patient-specific lattice constructs are complex and fragmented, with unclear requirements for documentation, testing, and long-term follow-up. Recent clinical studies involving tailored porous implants emphasize the variability in workflows and follow-up procedures, stressing the necessity for prospective registries and standardized reporting in order for PSLIs to progress beyond experiences limited to single centers.

CT and μCT workflows still face technical and biological constraints. Clinical CT resolutions limit accurate representation of thin cortical shells, trabecular orientation, and residual bone quality at the defect edges. Segmentation remains operator-dependent in many studies, with limited reporting on inter- and intra-observer variability. Current CT-model pipelines typically capture gross geometry and defect location but only coarsely approximate local bone quality, vascularity, and soft-tissue envelopes. Newer large-animal and clinical studies are beginning to distinguish peri-implant from intraporous bone formation using serial CT/μCT, but there is still no agreement on which metrics (e.g., BV/TV, BIC/BII, regional density maps) should guide design decisions. Future research should go beyond using simple masks of cortical and trabecular bone. We need to create detailed maps that show density, directionality, previous implants, and areas of dead tissue. These maps can help us design personalized solutions for lattice stiffness, fixation methods, and porosity levels.

Lattice design in long-bone reconstruction is still far from standardized. Studies explore a wide range of unit-cell topologies, gradient strategies, and infill patterns but often report only a few design variables and response metrics. Recent work on specific lattices (for example, distal femur designs tuned to target interfacial strain windows) and modular or LEGO-shaped scaffolds demonstrates how architecture, topology, and assembly can be tailored to anatomical regions and resection patterns, but these concepts are still in early, exploratory phases and lack comparative data across sites and defect types. There is a clear need for common reporting standards for lattice parameters (unit-cell type, cell size, orientation, density gradients, surface treatments) and for defect description (location, length, percentage of segment removed, fixation method), systematic exploration of the design space using Design of Experiments (DOE), or surrogate-based optimization, rather than ad hoc parameter choices—libraries of reference designs for typical femoral and tibial defect scenarios, which can be adapted to individual anatomy instead of starting from scratch for every case of distal femur, proximal tibia, and diaphyseal segments. Future work should couple parametric CAD frameworks with automated constraint checking (resection margins, fixation footprint, neurovascular safety windows) to avoid failure-prone designs and reduce iteration time.

Most numerical studies still rely on simplified boundary conditions, linear material models, and static loading scenarios. While such models are valuable for ranking design variants, they may not capture complex in vivo mechanics such as muscle forces, joint contact, multiaxial cyclic loading, and time-dependent bone remodeling. There is also limited systematic validation of FE models against experimental or clinical data, especially for fully implanted constructs under physiological loading paths. Some recent mechanobiology-guided frameworks use preoperative FE to target specific strain ranges at the bone–implant interface, but these remain isolated examples rather than a field-wide standard. Key directions include multi-scale simulations that link unit-cell mechanics, lattice-level behavior, and whole-limb load transfer; incorporation of nonlinear, damage; and fatigue models suitable for lattice architectures, including crack initiation and propagation in thin members, quantification of uncertainty due to image noise, segmentation variability, manufacturing tolerances, and patient-specific loading, moving from single best-guess simulations to probabilistic analyses, along with closer coupling of simulation with experimental and clinical readouts (e.g., strain gauges, motion analysis, serial CT) to iteratively validate and refine digital twin models of segmental reconstructions.

Experimental datasets remain challenging across three domains: mechanical tests (E_mech_), in vitro biology (E_bio_), and in vivo validation (V). Mechanical testing often uses simplified methods or surrogate defect models, with loading modes that only partially mimic the combined bending, torsion, and compression seen in femoral and tibial segments. In vitro cell studies typically focus on early adhesion, proliferation, and osteogenic markers, whereas long-term mineralization, vascularization, and immune responses in architected lattices are less frequently reported. In vivo work is still dominated by small-animal or simplified large-animal models that do not fully reflect the complexity of challenging human segmental scenarios.

Recent large-animal studies and early clinical series suggest that stable interface fusion between bone and porous titanium can sometimes provide durable function even without complete bone-to-bone bridging through the lattice, but the minimal amount and distribution of intraporous bone needed for long-term safety remain unknown. Future research should therefore prioritize standardized mechanical test protocols tailored to segmental long-bone reconstructions, including multiaxial fatigue and failure testing of full constructs (implant, fixation, and bone surrogate); longitudinal in vivo studies that correlate lattice geometry and stiffness with callus formation, bridging patterns, and remodeling at both peri-implant and intra-lattice regions, using CT/μCT metrics; and comparative studies against current standards of care (plates, nails, cages, distraction osteogenesis) to demonstrate not only feasibility but clear clinical advantage, including scenarios with and without bone grafting or biologics.

Additive manufacturing introduces process-specific challenges, including build defects, anisotropy, surface roughness, and variability between batches or printers. Many studies report nominal process parameters but provide limited information on process monitoring. For lattice implants with thin features and complex internal geometries, undetected defects or local deviations in strut thickness can dramatically alter mechanical performance and fatigue life. The emergence of new low-modulus titanium alloys and surface-functionalized lattices further increases the need for rigorous, standardized quality control.

Future work should integrate in-process monitoring with feedback into design and acceptance criteria; innovative nondestructive evaluation methods like μCT, ultrasound, and thermography to design lattice structures, ensuring reliability while adhering to clinically acceptable timelines; robust design approaches that account for manufacturing variability, ensuring safety margins even in the presence of realistic defects; and clear qualification and re-qualification protocols for printers, powders, and post-processing approaches used to fabricate CT-planned lattice implants intended for load-bearing long-bone applications.

Although fatigue/cyclic loading and time-dependent remodeling are repeatedly highlighted as key translational needs, their inclusion in the reviewed evidence base remains limited and cannot be quantified. Across the included studies, only one study reported an experimental cyclic loading protocol, implemented as a staged construct-level test (nine stages, 20,000 cycles per stage at 2 Hz; total 180,000 cycles; load increased from 0.5–1.0 × BW to 0.5–5.0 × BW) [11]. We did not identify fatigue-to-failure characterization (e.g., S–N curves, run-out definitions, R-ratio reporting, or ≥10^6^ cycles), and we did not find FE fatigue or damage-accumulation modeling used for long-bone segmental reconstruction in the included set (e.g., [1,63]). Longitudinal remodeling or follow-up outcomes (≥2 timepoints) were reported in five studies, typically as short-term preclinical monitoring (weeks) using radiography μCT or as longer clinical follow-up (months). Several papers explicitly acknowledge fatigue as a concern or limitation despite not performing fatigue testing [62,63]. Overall, this quantitative summary indicates that cyclic/fatigue validation is largely absent, whereas remodeling is more commonly assessed as an outcome but is rarely coupled with cyclic mechanical testing or time-dependent FE remodeling simulations.

Repeated loading introduces failure modes that static or quasi-static evaluations often miss, and these consequences matter for the construct’s long-term function. In lattice implants, stress and strain tend to concentrate at geometric discontinuities—strut junctions, curvature transitions, and notch-like surface features—including manufacturing-induced roughness and as-built defects, which makes these regions the most likely sites for crack initiation and progressive strut fracture under cyclic loading. Importantly, stiffness can start to degrade even before any obvious macroscopic fracture, and as struts fracture or locally deform, the lattice gradually becomes less stiff, shifting load transfer away from the intended porous region toward the fixation hardware and surrounding cortical bone. That redistribution can reduce intended load sharing, increase stress shielding, overload screws and plates, and alter interfragmentary motion in ways that undermine the mechanical environment required for bridging. At the same time, many FE studies assume perfectly bonded or no-separation interfaces, which can hide clinically relevant failure processes; in reality, small amounts of slip, fretting, and micromotion at the bone–implant interface can persist when osseointegration is incomplete, enlarge contact gaps over time, reduce the effective contact area, and further amplify micromotion—progressively driving interface loosening rather than appearing as a single sudden event. Taken together, these mechanisms explain why static, perfectly bonded simulations may overestimate long-term stability even when they show “safe” stress levels under a single load case, and they motivate reporting interface definitions and, where possible, adding cyclic loading protocols and time-dependent measures such as stiffness retention (or stiffness drop) and interface micromotion/peri-implant adaptation instead of treating mechanical performance as time-invariant.

Across the literature, data are often sparse, diverse, and not easily reusable. This limits the development of predictive or AI-based tools that could support clinicians in choosing defect-specific implant strategies (plate-and-lattice, nail-and-lattice, standalone cage, etc.). There is a substantial opportunity to build shared databases that link imaging, design parameters, simulation outputs, experimental results, and clinical follow-up for femoral and tibial segmental reconstructions. Recent work on mechanobiology-guided design and graft-free porous implants illustrates the potential value of such integrated datasets, but they are currently confined to individual centers or trials. In the future, data-based models could propose candidate lattice designs and fixation strategies given a new CT scan and defect classification; estimate the risk of mechanical failure, nonunion, or implant loosening under different design choices and rehabilitation protocols; and guide personalized exchange between stiffness, porosity, and biological potential, rather than relying solely on expert intuition.

Ultimately, the transition from experimental prototypes to routine clinical use will depend on demonstrating reliability, reproducibility, and cost-effectiveness. Bridging this gap requires coordinated efforts across disciplines: surgeons, radiologists, engineers, materials scientists, regulatory bodies, and industry partners. Prospective clinical registries for CT-planned lattice implants, standardized outcome measures (union rates, time to weight bearing, revision risk, patient-reported outcomes), and cost–benefit analyses will be crucial. Long-term follow-up of graft-free porous reconstructions, including cases with tumor or infection, is especially important to clarify indications, contraindications, and the need for adjunctive therapies such as anti-infective or anti-tumor coatings.

In summary, CT-planned, lattice-based reconstructions of femoral and tibial segmental defects hold clear conceptual advantages over historical approaches, but key obstacles in workflow integration, design standardization, simulation validation, experimental evidence, manufacturing quality, and data infrastructure must still be overcome. Addressing these challenges will define the next phase in the evolution from Ilizarov-era constructs to mature, clinically robust, patient-specific lattice implants.

## 8. Conclusions and Outlook

This paper, Part 2 of a two-part review series, examines patient-specific lattice implants (PSLIs) from initial design through translational application. Building on the defect- and fixation-based mapping presented in Part 1, this review reorganizes the literature along the workflow from CT or μCT acquisition and segmentation, through lattice and material selection, to numerical modeling, mechanical testing, in vivo validation, and early clinical use. By linking anatomical site, defect morphology, imaging workflow, implant concept, and methodological evidence level (S, E_mech, E_bio, V, C), this paper demonstrates how decisions made at each stage of the CT-to-implant pipeline influence mechanical performance, biological response, and clinical feasibility.

Several consistent patterns emerge. Imaging and segmentation remain the starting point for all PSLI concepts, yet acquisition, reconstruction, thresholding, and HU-based material mapping protocols vary widely and are often insufficiently reported. Similar variability is observed in the transition from segmented anatomy to CAD models and FE simulations, where choices related to region definition, cortical–trabecular treatment, interface assumptions, and loading conditions substantially affect the reported outcomes. At the same time, the design space of lattice and mixed-material constructs is extensive—ranging from fully porous cages and functionally graded structures to composite and scaffold-guided regeneration concepts—while only a limited subset has been investigated under realistic loading and healing scenarios. Overall, the literature remains dominated by numerical studies and quasi-static mechanical tests, whereas fatigue behavior, long-term large-animal data, and harmonized clinical follow-up are comparatively limited.

Importantly, the reviewed studies show that PSLIs have progressed beyond only experimental concepts. CT-planned titanium cages used within Masquelet protocols, topology-optimized and TPMS-based distal femur reconstructions, modular scaffold systems, and degradable bioceramic implants have demonstrated that architected porosity can be combined with plates, nails, or external fixation to restore alignment and enable progressive loading in complex femoral and tibial defects. When imaging, design, manufacturing, and validation are integrated into a workflow, these constructs can achieve stable fixation and support early healing, even in cases involving infection, tumor resection, or large segmental gaps.

Taken together, the findings presented in this second part suggest that future progress will depend less on proposing new lattice topologies and more on establishing robust, traceable workflows from DICOM data to implanted constructs. This includes clear reporting of imaging and segmentation protocols, reproducible strategies for selecting lattice families and gradients for specific defect patterns, validated numerical models linking unit-cell behavior to whole-limb mechanics, standardized mechanical and biological testing tailored to segmental defects, and additive manufacturing process control that connects monitoring data to acceptance criteria for thin-walled lattice regions. When these elements are integrated into interoperable planning environments and supported by prospective registries capturing union, function, complications, and reoperations, PSLIs can be evaluated alongside established reconstruction strategies such as bone transport, vascularized grafts, and megaprostheses.

Read together with Part 1, this paper provides two complementary perspectives: one focused on defect patterns and fixation strategies, and the other on the design and validation of patient-specific lattice implants. Integrating these perspectives outlines a clear path toward transforming isolated, center-specific applications into reproducible treatment approaches. With consistent imaging quality, sound lattice design, reliable manufacturing, and systematic long-term follow-up within clinically acceptable timelines, patient-specific lattice implants have the potential to evolve from selected case solutions into a standard option for challenging femoral and tibial segmental reconstructions.

## Figures and Tables

**Table 1:** CT- and μCT-based imaging-to-model workflows used across the included studies.

Year, Ref [n]	Imaging-to-Model Workflow (CT/μCT; Key Steps)
2013, [1]	CT of standardized composite femur (4th-gen large left Sawbones); matrix/pixel/slice not reported; segmentation not specified; CT-based HU mapping from composite bone.
2014, [70]	No patient CT model pipeline; bench μCT of SLM Ti implants (thresholds 77–255/85–255/96–255); compute porosity, pore and strut sizes; no DICOM segmentation/mirroring; no CT-based CAD.
2015, [65]	No CT (standardized 3rd-generation composite femur from Biomedical Research Community), segmentation N/A; geometry used directly (no mirroring); canal prepared by drilling/reaming via Boolean ops; CT-based HU mapping not used.
2018, [68]	CT-based design, matrix/pixel NR, slice thickness NR; CAD generated from 3D-CT by manufacturer (DePuy Synthes); no mirroring stated; radiographs throughout and CT for clinical follow-up; segmentation/stacking/export/software NR.
2018, [67]	CT (healthy adult femur, DICOM), matrix/pixel/slice not reported, segmentation in Mimics (ROI thresholding; healing of crumbled parts; noise reduction), export point cloud from Mimics to SolidWorks and convert surface to solid (target point cloud size set to “random”), HU measured in Mimics with mapping used for porosity and modulus.
2019, [15]	No CT/DICOM (composite femur CAD from manufacturer), optical white-light scan of cortex and core (3D3 Solutions HDI Advance R3) with FlexScan 3D, surfaces generated in Rapidform XOR3 and assembled in SolidWorks 2016 (Sawbones part 3908), segmentation N/A, registration/mirroring N/A, DIC system (GOM ARAMIS) used for full-field strain validation.
2019, [5]	CT (average adult human femur), matrix/pixel/slice not reported, 3D reconstruction in Mimics (Materialise) → Geomagic Studio to create femur CAD, Boolean of arrayed unit cells with selected femur region to build reconstructed scaffold model, no mirroring reported, export as CAD for printing/analysis; CT-based HU mapping not reported.
2019, [69]	CT (human femur mid-diaphysis; matrix/pixel NR; slice thickness NR), DICOM export, Mimics (segmentation and 3D reconstruction; version NR), STL export.
2019, [2]	High-resolution CT of temporary PMMA-spacer construct (stage-1 Masquelet) for virtual surgical planning; matrix/pixel NR; slice thickness NR; surgeon-led VSP to define approach, screw trajectories, and plate/IM devices; contralateral limb mirrored to restore native contours; CT data sent to manufacturer (4Web Medical) to CAD/3D-print; follow-up with routine radiographs (measured in AGFA IMPAX) and CT at 9–12 months; export/stacking formats NR (planning CT targets spacer construct rather than direct bone segmentation).
2020, [9]	In vivo X-ray μCT (Skyscan 1176, Bruker): pixel 35.4 μm, 65 kV, 373 μA, 1 mm Al filter, 0.7° rotation, 58 ms; reconstruction (CTAn) → 3D femur model in Simpleware ScanIP; in vitro/ex vivo μCT (Skyscan 1176): pixel 17.7 μm, 80 kV, 300 μA, Cu+Al filter, 0.5° rotation, 100 ms; reconstruction (NRecon v1.6.8.0); quantitative analysis in CTAn with grayscale thresholds (bone = 63, HA = 120); outputs reported as total bone volume and intraporous bone volume.
2020, [3]	CT (bilateral femurs), matrix/pixel/slice not reported, cortical/cancellous contours extracted from CT slices and converted to 3D solid models in Creo Parametric v5.0, intact left femur mirrored (1 cm proximal/distal to fracture) to restore right femur via Boolean; PSI geometry derived from restored anatomy; exported to FE model with nonlinear contact (solver not specified).
2021, [14]	CT (single healthy adult femur), 512 × 512 matrix, pixel 0.6445 mm, slice 1 mm, Mimics v17 threshold/region-grow, no mirroring (section-based analysis), direct to ANSYS for meshing/FE and STL for AM, CT-based HU–density-E mapping applied.
2021, [6]	In vivo radiography (serial X-rays) and μCT over 12 weeks for rat femoral CSBD; scanner/model not reported; μCT used to assess scaffold porosity/morphology and to generate μCT reconstructions of implanted gyroid-sheet scaffolds; specific voxel size and segmentation thresholds not reported; image exports/software for μCT analysis not specified.
2021, [64]	CT, matrix/pixel/slice not reported, 3D Slicer 4.11 to convert DICOM to 3D model, STL repair/prep in Autodesk Meshmixer, no mirroring, HU-E mapping not reported; SLA prints (Anycubic Photon S) used for validation.
2021, [4]	CT, μCT (Inveon MM, Siemens) 20 μm voxel for ex vivo analysis, segmentation for FE in Mimics Research 20.0, μCT 3D recon in Inveon Research Workplace, HU threshold 1000-3885 to separate new bone from implant, ROIs defined as peri-implant (2 mm peripheral ring) and intraporous (within pores), clinical follow-up included μCT/histology at implant–bone interface; matrix/pixel/slice for clinical CT not reported; mirroring not reported; HU-E mapping not stated.
2021, [4]	Clinical CT-based design, implants designed from 3D-CT data (matrix/pixel NR, slice thickness NR), variable CT/X-ray follow-up (e.g., immediate to 36 months); Sheep: axial spiral CT (Siemens SOMATOM Definition Flash 64), 120 kV, 200 mA, FOV 21 cm, slice thickness 3 mm; μCT for endpoint morphometry (Siemens Inveon MM), nominal resolution 20 μm, segmentation/3D reconstruction in Inveon Research Workplace, HU threshold 1000–3885 for new bone, ROIs: peri-implant 2 mm belt and intraporous; CT to CAD/FEA by Mimics Research 20.0; stacking/export formats NR; mirroring NR.
2022, [7]	CT/DICOM (adult femur; standard clinical scan), matrix/pixel/slice not reported, MIMICS 18.0 region-grow 230–1883 HU, STL-FreeCAD-IGES-SpaceClaim 2021 R1 section cut to create segmental defect, HU-E mapping not stated.
2022, [11]	Patient CT (distal lateral femur, osteosarcoma), matrix/pixel/slice not reported, segmentation tool not reported, defect defined anatomically with rule H = Y/3 above the epiphyseal plate (no mirroring), CAD from CT images.
2023, [62]	CT, DICOM 3.0; matrix/pixel/slice not reported, segmentation in Mimics 21.0 (threshold by gray values), surface repair in Magics 22.0, contralateral mirroring used to reconstruct the defect site, osteotomy length set to 90 mm by prior clinical reference, export of repaired femur/PSI geometry for FE analysis (solver not specified), HU-E mapping not reported.
2023, [8]	μCT (Scanco μCT 100) of printed Ti scaffold to verify geometric features, 90 kVp, 200 μA, 18 W, integration 140 ms, nominal resolution 4.9 μm, images reconstructed/visualized in Scanco workstation (no patient DICOM modeling); no mirroring; CAD did not originate from CT (parametric design).
2023, [17]	CT, matrix/pixel not reported, slice distance 2 mm, 1816 cuts, DICOM export; Mimics 10.01 density-based segmentation to primary 3D model; no mirroring stated; slicing in Cura (LulzBot Edition v3) for AM.
2023, [10]	CT (ovine metatarsus, pre-op planning), DICOM, voxel 0.115 × 0.115 × 0.600 mm, InVesalius interactive thresholding + 3D reconstruction (stacking); defect templating by cropping 13 mm bone segment, medullary cavity fill, addition of coupler (Ø4 × 2 mm) and graft hole (Ø4 × 10 mm); no mirroring; export format NR; microstructure optimized numerically in prior work (not CT-derived). Follow-up CT for analysis: 300 μm/voxel, manual segmentation in InVesalius, contralateral limb co-scanned as control; HU-BMD calibration using QRM-BDC/6–200 phantoms (0–0.8 g HA/${\mathrm{cm}}^{3}$); outputs: callus TV, CSA, BMD over time.
2023, [13]	Ex vivo X-ray CT (XCT) on explanted sheep implants with surrounding bone; voxel 6.8 μm, 120 kV, 120 μA, 3142 projections, 1000 ms/projection, 2 × averaging, 2 h scan; 3D stacks reconstructed in XCT workstation; segmentation via a 3-stage ML workflow: Ilastik pixel classification to generate labels to two custom 2D U-Nets; classes separated: metal, bone, pores, osteoid, metallic grains; purpose: quantify BII (<10 μmm) and BIC (95%); no patient CT→CAD or mirroring (QC/analysis only); export/software for CAD NR.
2024, [16]	CT (adult New Zealand White rabbit tibia), matrix/pixel not reported, slice thickness not reported, Meshmixer 3.3 for extraction/smoothing/repair of left tibia, Geomagic Studio for SolidWorks 2017 for parametric surface patches, no mirroring, material mapping not stated, models prepared for SLM prototype and bench tests.
2024, [66]	2D medical X-rays (no CT), matrix/pixel/slice not reported, external femur surface via reverse engineering from 2D images, segmentation N/A, no mirroring, algorithmic 3D reconstruction from 2D, export to CAD assembly of modular “BoneBricks” blocks, material mapping not applicable, bench/prototyping only.
2024, [12]	CT-based femur model (ENOVO-186 dataset), matrix/pixel NR, slice thickness NR; segmentation/stacking NR; geometry modeled from CT image; CAD in Creo Parametric ν5.0; validation CT used to quantify model/bone volumes (animal), export formats NR, mirroring NR.
2025, [63]	CT (NIH Visible Human, male right femur), 512 × 512 matrix, 1 mm slices; segmentation in Amira–Avizo (Python script used to record HU), surfaces exported as STL; smoothing/solid conversion in Geomagic Freeform and Geomagic Wrap; no mirroring; HU–density-E material mapping applied per element with manual mapping in ANSYS Workbench (triangulation, volumetric transfer).

NR—Not Reported; BMD—bone mineral density; CSA—cross-sectional area; CTAn—CT-Analyser software for micro-CT image analysis; NIH—National Institutes of Health; ROI—region of interest; SLA—stereolithography; TV—total volume; ML—machine learning; BII—bone–implant contact; BIC—bone–implant interface.

**Table 2:** Lattice design parameters, gradient strategies, and CAD tools reported in femoral and tibial scaffold studies.

Year, Ref [n]	Lattice	UC (mm; X × Y × Z)	T (mm)	PS (μm)	RD/ Porosity (%)	SA/VR (${\mathrm{mm}}^{-1}$)	Gradient (Type)	Gradient Driver	Design Objective(s)	CAD Software
2013, [1]	Strut (regular open-cell; cylindrical scaffold)	-	0.3–0.8	800/1100 /1500/1800	61–81 (eff.); 64–93 (scaf.)	—	None (constant RD)	Rule-based parametric sweep	E-match; SS-reduce; minimize gap Δ d; $\sigma_{\mathrm{vM}}\;<\;660$ MPa	NR.
2014, [70]	Strut-based lattice (dodecahedron unit cell)	0.5 × 0.5 × 0.5	0.12/0.17/0.23 (strut diameter)	500 (nominal); ≈560–610 (μCT)	84/78/68 (porosity)	NR	None (uniform lattice; three stiffness groups)	- (Stiffness tuned by global porosity, no spatial gradient)	Vary implant stiffness to study load transfer	NR
2018, [68]	Strut-based honeycomb Ti-mesh	cylindrical scaffold: 20 × 40 (*diameter* × *length*); central canal 10 (diameter); strut length 7	1.2/1.6 (for soft/stiff scaffold)	NR (macrochannels in mm range; exact values not reported)	NR	NR	None (uniform honeycomb architecture N/A (global stiffness tuned via FE, not spatially graded)	Mechanobiologically optimize Ti-mesh stiffness to minimize stress shielding, avoid mechanical failure, and create a favorable strain environment for bone regeneration	Proprietary manufacturer CAD (DePuy Synthes; exact software NR)	NR
2018, [67]	Strut (polyhedral unit-block: cuboctahedron; hexahedron; truncated hexahedron; truncated octahedron)	NR (unit-cube basis)	NR (beam thickness via PS:BT)	NR	5–60	NR	RD gradient (functionally graded scaffold)	Rule-based (vary PS:BT to set porosity profile)	E-match; SS-reduce; open structure for ingrowth	Mimics; SolidWorks; Pro/Engineer (Creo)
2019, [15]	Strut (orthogonal grid; square/ hexagon)	NR	NR	NR	60, 70 (small cubes); 60 (cyl.)	NR	None	N/A	Match Eeff, strain-energy; orthotropy (validate simplified homogenization)	SolidWorks 2016
2019, [5]	Spherical pore; TPMS gyroid; Topology-optimized	2.4 × 2.4 × 2.4	NR	SP: 1110; G/TO: NR	70	NR	None	N/A	Maximize initial mechanical performance at fixed porosity (70%); degradation-resilient retention	Mimics (Materialise Inc., Leuven, Belgium) and Geomagic Studio (Geomagic Inc., Cary, NC, USA).
2019, [69]	Honeycomb scaffold (square/triangular pores)	Scaffold block: 32 × 25.5 × 13.5	0.2032	1250 (square/tri- angular pores)	≈68–83 (designed layer blocks); ≈82 (femur mid- diaphysis scaffold)	NR	None (uniform pore architecture per pattern)	N/A	Design ABS FDM scaffolds with controlled pore size and porosity to achieve cortical bone-like stiffness and strength in a femur mid-diaphysis segment; study influence of FDM process parameters on structural modulus and compressive strength	CATIA and Insight (Stratasys Fortus 360mc)
2019, [2]	Patient-specific truss-type titanium cage (additively manufactured)	Anatomical cage spanning femoral segmental defect; truss microarchitecture not specified	NR	NR	NR (highly porous cage intended for large-volume bone graft packing)	NR	None (no explicit porosity or stiffness gradient; geometry matched to defect)	N/A	Custom 3D-printed titanium cage used with the Masquelet technique to reconstruct massive segmental femoral defects, restore alignment/length, provide immediate mechanical stability, and create a contained space for large volumes of bone graft within an induced membrane	Manufacturer-specific tools (exact CAD software NR)
2020, [9]	TPMS gyroid (GP); hybrid: gyroid + cortical-like outer shell (GPRC)	0.81 (equivalent unit cell)	0.18–0.23 (outer shell); internal NR	430 (free- moving sphere)	GP: 60; GPRC: 43	NR	None (GP); morphology hybrid (shell and lattice) (GPRC)	Rule-based (fixed shell; not CT-guided gradient)	Fit 3 mm rat femoral defect; target pore for ingrowth (430 μm); add shell to boost handling/strength (HA)	ScanIP (Simpleware, UK)
2020, [3]	Grid mesh + solid-shell lattice (patient-specific)	10 × 10 with 1.5 mm thickness: surface mesh	1.5	NR	NR	NR	None	Rule-based (rounded angle mesh; patient-specific shell fit)	Lightweight, reduce stress shielding, promote osseointegration, protect graft, minimize stress concentrations	Creo Parametric v5.0 (PTC, Needham, MA, USA)
2021, [14]	Strut (simple cubic infill; hollow REVs)	NR (REV-based)	≥0.5 (variable)	NR	Variable (derived; NR exact)	NR	RD/wall-thickness gradient	CT-guided HU-E	E-match; SS-reduce; osseointegration-friendly	Mimics version 17
2021, [6]	TPMS-gyroid (sheet)	3 × 3 × 3; 6 × 6 × 6	0.30; 0.60	739; 1076	CAD 70; μCT 62.8–70.8	NR	None	-	Bone ingrowth; interface stability; pore-size effect; BMP2 carrier	NR
2021, [64]	TPMS (gyroid; primitive; diamond; lidinoid) + strut (Kelvin) + honeycomb	NR (scaffold cubes 20 × 20 × 20)	NR	NR	NR	NR	None (single lattice per specimen)	100–500	Compare mechanical performance across lattice types; assess effect of HA/CPP additive on E, UTS, $\epsilon_{b}reak$	Rhino 7
2021, [4]	Porous Ti6Al4V implant (microarchitecture NR)	NR	NR	NR	NR	NR	NR	NR	Immediate stability and implant–bone interface fusion; 3-point bending FEA validation.	Mimics Research 20.0.
2021, [4]	Open-cell, strut-based	NR	0.24–0.32	400–600	60–80	NR	None (uniform porosity; patient-specific outer shape only)	N/A	One-stage, bone-graft-free reconstruction of large segmental bone defects using individualized porous Ti implants designed for immediate mechanical stability and long-term implant–bone interface fusion in limb, spinal, and pelvic defects	Mimics Research 20.0 software (Materialise, Belgium); EBM S12 system (Acram AB, Sweden)
2022, [11]	Surface lattice (windowed grid) + inner circular holes	5 × 5 —(outer grid); 10 × 10 for larger bone	2 (shell); 2 (stent width)	5000; 5000	N/A	NR	None	Rule-based patient-size scaling (window distances; Y/3)	Minimize stress/shielding; enable graft fill	Bone ingrowth; lightweight via topology optimization (retain 15% vol), Geomagic Studio and PTC Creo
2023, [62]	Hybrid: strut (TBC, BCC) + trabecular mimic (stochastic)	NR	NR	NR	55 (TBC, bearing); 65 (I-structure, transition/cancellous); 86 (trabecular-mimic, interface)	NR	Morphology + porosity (stiffness) gradient (module-based)	Rule-based by region (bearing vs. interface)	Increase interface bone strain; reduce stress shielding; keep relative displacement less than 150 μm; more uniform stress under daily loads	Mimics 21.0; Magics 22.0
2023, [8]	Strut-based open-porous ATS blocks (NP/SP/UP)	8 × 8 × 5.8	0.2–0.5	400–800 (square channels)	50% (SP), 62% (UP)	NR	None (discrete NP/SP/UP designs)	N/A	Modular LEGO-like Ti scaffold blocks for intraoperative, patient-specific assembly; comparison of NP/SP/UP porosity and surface texturing for mechanical performance and osteogenesis	Fusion 360 (Autodesk); Magics (Materialise)
2023, [17]	No lattice (solid anatomical femur model)	-	-	N/A (solid; no pores)	100 (solid infill)	NR	None (no gradient)	N/A	Develop a 3D-printed CF-PEEK femur model with mechanical behavior close to human femur for FE validation and bone-plate biomechanical testing	Mimics 10.01; SOLIDWORKS 2020
2023, [10]	Robocast rod-based bioceramic lattice (layer-wise orthogonal rods)	Subject-specific defect-filling scaffold for 15 mm ovine metatarsal gap	-	360.8	59.3/≈40.7	≈5.77	None (uniform microarchitecture)	N/A	Numerically optimized inner architecture to maximize porosity, specific surface area, and pore size for cell diffusion, adhesion, and proliferation while maintaining mechanical integrity under physiological loads in the ovine metatarsus	InVesalius (Renato Archer Information Technology Center, Amarais, Brazil)
2023, [13]	Strut-based cubic octahedron lattice (Ti–19Nb–14Zr cylindrical implants for sheep tibia/ metatarsus)	0.9 × 0.9 × 0.9/1.2 × 1.2 × 1.2	NR	350/450 (octahedron diagonal within 900/1200 μm cells)	NR	NR	None (uniform lattice; three designs differ only by lattice height and side closure)	N/A (no spatial gradient; access limited by side walls and top/bottom closure)	Investigate in vivo bone progression and bone–implant contact in and around Ti–19Nb–14Zr lattice implants as a function of cell size (900/1200 μm), lattice accessibility (open/side-closed/half-side-closed cylinders), and implantation site (tibia vs. metatarsus) using 3D XCT and ML-based segmentation	CAD software: NR (lattice cylinders designed as 6 mm diameter implants)
2024, [16]	Hybrid (solid-shell and lattice; Ti cage (higth = 5 mm) and HAp core)	NR	NR	NR	50%, 70% (single); radial 50→70%	NR	RD gradient (radial); material gradient	Rule-based (higher porosity near bone plate; decreasing per layer)	SS-reduce; uniform stress; protect HA; stable fixation	SolidWorks 2017; Geomagic for SolidWork; Meshmixer
2024, [66]	Strut (extrusion path zigzag/spiral); modular “bone bricks”	NR	NR	NR	NR (module-dependent)	NR	Porosity gradient (module palette assembly)	Rule-based, anatomy-driven from 2D images; module parameters (division numbers) yield consistent pore layout	Fit-PSI; fast, low-cost modular fabrication; adequate porous morphology for ingrowth	NR (custom algorithm)
2024, [12]	Strut-based cuboctahedron lattice	2 × 2 × 2	0.6–1.0 (pillar diameter)	NR (cuboctahedron voids on mm scale; not explicitly quantified)	NR (lattice stiffness varied via pillar diameter/alignment; porosity not tabulated)	NR (only relative surface area vs. alignment reported)	None (uniform lattice; different global variants by angle and pillar diameter)	N/A (no explicit spatial gradient driver; parameters chosen via FE strain response)	Parametrically optimize cuboctahedron lattice alignment angle and pillar diameter to generate target interfacial bone strain (∼4000 μϵ) and stimulate osseointegration in large distal femur defect reconstruction (OLS implant)	ANSYS Workbench 2022R2 (Material Designer module)
2025, [63]	Strut (pillar lattice; designs A/B/C)	6 × 6 × 6	1.5; 2.0; 2.5 (pillar ϕ); fillet r = 1	NR	NR	NR	None	Rule-based (pillar-ϕ sweep; edge fillets to reduce stress concentration)	SS-reduce; E-match (deformation = intact); limit $\sigma_{\mathrm{implant}}$; 880 MPa; Fit-PSI	ANSYS DesignModeler

ATS—assemblable titanium scaffold; BCC—body-centered cubic lattice; BMP-2—bone morphogenetic protein-2; CF-PEEK—carbon fiber-reinforced polyether ether ketone; E—elastic modulus (Young’s modulus); G/TO—bone growth/turnover; HA/CCP—hydroxyapatite/calcium pyrophosphate composite; NR—not reported; OLS—open lattice structure; PS—pore size; SP—single-porosity; SS—stress shielding; T—thickness; TBC—trabecular bone cylinder; UC—unit cell; UP—uniform porosity; UTS—ultimate tensile strength.

**Table 3:** Manufacturing routes, process parameters, and characterization protocols for the implant and scaffold constructs included in this review.

Year, Ref [n]	Initial Material	Manufacturing Method	Manufacturing Device/Company	Primary Process Parameters	Post-Processing	Final Part/Section in Bone	Characterization Techniques
2013, [1]	Ti6Al4V	SEBM	–	–	–	Nonporous tensile samples and custom-made open-porous compression test samples	–
2014, [70]	Ti6Al4V ELI grade 23, 25–45 μm	SLM	Layerwise NV (Leuven, Belgium)	Yb:YAG fiber laser; Ti base plate; LP = 42 W; LT = 30 μm; ν = 260 mm/s	EDM	Highly porous Ti implants for segmental bone defect; unit cell type: dodecahedron; strut sizes = 120, 170, 230 μm; pore size = 500 μm	–
2018, [68]	Ti alloy	Laser sintering	Manufactured by DePuy Synthes company (Warsaw, IN, USA)	–	–	Ti-mesh scaffolds	BSE-SEM for bone generation study within the scaffold
2019, [15]	VeroWhitePlus (RGD835) photopolymer (liquid resin)	Material jetting (PolyJet)	PolyJet 3D printer, Objet Eden260VS (Stratasys, Minnetonka, MN, USA)	16 μm layer resolution	–	Rectangular and cylindrical scaffolds with square and hexagonal patterns and 60–70% porosity	DIC
2019, [5]	PLA–β-TCP-HA composite slurry (ratio 2:1:1)	Extrusion bioprinting	Bio-printer (Regenovo Biotechnology Corp., Hangzhou, China)	Printing speed 0.20 mm/min; P = 0.25 MPa; T = 25° C; *⌀* printing needle = 610 μm	–	Biocomposite scaffolds with spherical pore, gyroid, and topological architecture	SEM analysis to observe the appearance of samples before and after degradation
2019, [69]	ABS polymer	FDM	Fortus360mc 3D printer (Stratasys, Minnetonka, MN, USA)	Nozzle tip T10; $LT_{\min}\;=\;0.127$ mm; four raster laydown patterns; minimum slice thickness = 0.127 mm; raster width = 0.2032 mm; maximum air gap between roads = 1.27 mm	Support structure of water-soluble SR30 material removed	Scaffold for femur bone segment	Porosity assessment by unit cube and relative density methods
2019, [2]	Ti6Al4V	Most likely EBM (manufactured by 4Web Medical; Frisco, TX, USA)	EBM machine (Arcam AB, Mölndal, Sweden)	–	–	Patient-specific 3D-printed titanium cages	–
2020, [9]	HA powder made into a slurry suitable for 3D printing	Indirect AM: material jetting (Inkjet DoD) for wax molds and further impregnation with slurry	3Z Studio (Solidscape, Multistation, Saint-Malo, France); adjustable LT = 6,12,19,25 μm)	LT = 25μm; printing orientation vertical (implant length along printer’s Z axis); uniform 14% expansion of initial CAD to accommodate sintering shrinkage	Overnight drying at RT; debinding at 500 °C; sintering in air at 1200 °C with heating rate of 4 °C/min for 2 h	HA implants with gyroid porosity reinforced by a cortical-like outer shell	ImageJ freeware (National Institutes of Health, Bethesda, MD, USA) freeware for porosity analysis (micropore shape and size); SEM for surface macro-topography; FTIR and XRD to analyze phase stability of the initial HA
2020, [3]	Ti alloy (∼30 μm particle size)	LPBF	AM400 (Renishaw, Wotton-under-Edge, Gloucestershire, UK)	–	Printed implant surface sandblasted, followed by a specific cleaning protocol	Patient-specific 3D-printed titanium implant	–
2021, [14]	Ti6Al4V; (4–40 μm particle size)	DMLS	EOSINT M280 (EOS GmbH, Krailling, Germany)	Yb-fiber laser; spot size = 80 μm; ν = 1200 mm/s; HD = 0.14 mm	Stress relief heat treatment at 800 °C for 1.5 h in Ar gas chamber	Porous Ti6Al4V implant for greater trochanter, diaphysis, and epicondyle sections in bone	–
2021, [6]	Ti6Al4V (medical grade)	LPBF	3D Systems DMP ProX 320 (Rock Hill, SC, USA)	–	HIP to reduce residual stresses and improve ductility; EDM; microblasting	Gyroid-sheet topology lattice with 70% porosity and 740 or 1100 μm average pore sizes	ImageJ (NIH) and BoneJ plugin for pore size and wall thickness measurement
2021, [64]	(1) Biodegradable resin from soybean oil; (2) biodegradable UV-cured resin with 5% HA and 5% CPP	SLA	SLA 3D printer (Anycubic Photon, Shenzhen, China)	–	Specimens heated for 40 h at 230 °C before tensile testing	TPMS and FGLS scaffolds with 100–500 μm pore size	Displacement method (submersion) for porosity and density analysis
2021, [4]	Ti6Al4V	EBM	EBM S12 system (Arcam AB, Mölndal, Sweden)	–	Air-blasted and ultrasonically cleaned	Ti6Al4V porous implants with pore size 40–600 μm, strut diameter 240–320 μm, porosity 60–80%	–
2022, [11]	Ti6Al4V	LPBF	AM400 (Renishaw, Wotton-under-Edge, Gloucestershire, UK),	–	–	Bone scaffold implant	–
2023, [8]	Pure titanium (Ti grade II,$d_{50}\;=\;41\pm 2$ μm	SLM	SLM 250HL (SLM Solutions GmbH, Lübeck, Germany)	LT = 30 μm; outer contour: LP = 100 W, ν = 550 mm/s; inner contour: LP = 175 μW, ν = 833 μmm/s	One ATS set surface textured by double-acid treatment at elevated temperature for 15 min	LEGO^®^-interlocking design-inspired ATS with surfaces: native SLM and textured (double-acid-etched)	Imbibition analysis (absorption of liquid by solid scaffold); surface roughness by laser profilometer
2023, [17]	3DXTECH CF-PEEKfilament spool	FDM	Pratham∼5.0 3D printer (Make3D.in, Surat, India)	Single extruder; *⌀* support extruder = 400 μm; extruder nozzle temperature = 300 °C; build plate temperature = 120 °C; LT = 100–500 μm; print time = 12 h	–	Femur model	–
2023, [10]	Clinically proven 45 vol.% HA ink	Material extrusion (DIW)	3-D Inks robotic deposition device (Still-water, Tulsa, OK, USA)	–	Dried at 400 °C for 1 h; sintered at 1300 °C for 2 h; sterilized under high formaldehyde concentration at 60 °C and 75–100% relative humidity	Scaffold for 15 mm bone segment with 59.30% porosity, 5768.91 $m^{-1}$ specific surface area and 60.80 μm pore size	–
2023, [13]	Ti-19Nb-14Zr (at%), ZTM14N	LPBF	SLM 125HL (SLM Solutions GmbH, Lübeck, Germany)	Yb-fiber laser; LP = 200 W; LT = 30 μm; build plate temperature = 200 °C	Cleaning and sterilization	Lattice implants for tibia and metatarsal bones with three cylindrical designs and two main unit cell sizes (900 and 1200 μm) and diagonal cell sizes (350 and 450 μm)	He-pycnometry for bulk density and relative density
2024, [16]	Ti6Al4V	SLM	EOSINT M280 (EOS GmbH, Krailling, Germany)	Yb-fiber laser; $LP_{\max}\;=\;200$ W	Tested annealing conditions: 723–923 K and 40–240 min; optimal condition: 842.8 K, 77.6 min	Ti6Al4V scaffold with radial gradient porosity (50–70%) filled with HA for the critical-sized tibial defect	Nanoindentation to determine mechanical property variations of the annealed scaffold, including elastic modulus and hardness
2024, [66]	PCL filament	FDM	Commercially available 3D printer	Syringe temperature = 65 °C; *⌀* extrusion nozzle = 250 μm; zigzag scan strategy	Assembly of modular scaffold blocks by the surgeon	Scaffold structure for 67.60 mm distal bone defect and femur models	–
2024, [12]	Ti6Al4V	LPBF	AM250 (Renishaw plc, Wotton-under-Edge, Gloucestershire, UK)	Spot diameter = 75 μm; LT = 30 μm; LP = 100 W; ET = 60 μs; PD = 75 μm; HD = 20 μm	Complementary femur model fabricated using FDM polymer 3D printing	OLS with 69.8% porosity and non-lattice-structured solid implant; lattice unit cell: cuboctahedron	–

ATS—assemblable titanium scaffold; DIW—direct ink writing; EDM—electrical discharge machine; ET—exposure time; HD—hatching distance; HIP—hot isostatic pressing; LT—layer thickness; PD—point distance; RT—room temperature; SEBM—selective electron beam melting; *ν*—scanning speed, YAG—yttrium aluminum garnet.

**Table 4:** Finite element modeling strategies for patient-specific lattice and scaffold implants.

Year, Ref [n]	Material Law	Boundary Conditions/Loads	Software/Mesh	Outputs/Metrics	Validation	Key Mechanical Outcomes
2013, [1]	- Linear elastic - Isotropic bone - Homogeneous scaffold (E from tests)	- Distal femur fixed - Single-leg stance loading	Abaqus 6.10/C3D8	- Von Mises stress - Axial stiffness - Interfragmentary motion	- Uniaxial tests on porous Ti	- Higher stiffness: lower motion - Compliant designs: excessive deformation - Porosity–stability trade-off
2015, [65]	- Linear elastic - Isotropic bone and hardware	- Alternative distal fixation - Axial, bending, torsion	ADINA 8.9/NR	- Construct stiffness - Stress in nails and screws	- Comparison to literature data	- BC choice strongly affects stiffness - Simplified BCs distort load transfer
2018, [68]	- Linear elastic - Isotropic Ti, bone, callus	- Axial compression (1372 N) - Bending load (86 N)	NR	- Von Mises stress - Principal strain - Pore strain distribution	- FE-guided scaffold selection	- Soft scaffold: higher callus strain - Stiff scaffold: stress shielding
2018, [67]	- Linear elastic - Isotropic bone and scaffold	- Distal femur fixed - Axial compression	ANSYS/Hypermesh 2 mm tetra	- Stress distribution - Global displacement	- None (numerical only)	- Higher porosity: lower stiffness - Increased scaffold stress
2019, [15]	- Linear elastic polymer - Orthotropic homogenization	- Compression to 500 N	Abaqus 6.13/NR	- Effective modulus - Full-field strain	- Compression tests - 3D DIC	- Homogenized model matches detailed lattice - Reduced computational cost
2019, [5]	- Linear elastic composite - Isotropic behavior	- Uniaxial compression - Displacement-controlled	ANSYS/NR	- Von Mises stress - Effective stiffness	- Compression tests	- Gyroid and TO stiffer than spherical pores
2020, [3]	- Linear elastic bone - Ti implant - Healing-stage-dependent	- Distal femur fixed - Single-leg stance	ANSYS/NR	- Stress and strain - Articular displacement	- None	- Acceptable stresses across healing stages - Peak stress early post-op
2021, [14]	- HU-based heterogeneous bone - Linear elastic	- Distal constraint - Physiological loading	ANSYS/NR	- Construct stiffness - Bone strain	- Compression tests	- Stiffness-matched designs reduce mismatch
2021, [4]	- Linear elastic - Isotropic bone	- Three-point bending	Abaqus 6.4/NR	- Bending stiffness - Interface stress	- Ex vivo bending tests	- Adequate stiffness - Safe interface stresses
2022, [7]	- Linear elastic - Isotropic scaffold and bone	- Axial compression - Single-leg stance	ANSYS/Hex mesh	- Stress–porosity relation - Deformation	- None	- TPMS restores load transfer - Reduced stress concentration
2022, [11]	- Linear elastic - Isotropic bone and Ti	- Physiological joint loading	ANSYS 19.0/NR	- Stress and strain - Construct stiffness	- In vitro strain gauges	- Reduced stress shielding - Maintained stiffness
2023, [62]	- Linear elastic - Stiffness-gradient scaffold	- Distal fixation - Daily activity loads	NR/NR	- Stress - Interface micromotion	- None	- Gradients reduce stress peaks - Lower micromotion
2023, [8]	- Linear elastic Ti - Isotropic	- Distributed pressure - Fixed opposite face	NX 12.0/Tetra	- Stress - Deformation	- Compression tests	- Large safety margin below yield
2023, [17]	- Linear elastic - Bone, PEEK, CF-PEEK	- Femur fixed - load applied on the femoral head	ANSYS/5 mm mesh	- Stress - Deformation	- Compression tests	- CF-PEEK closest to femur response
2024, [16]	- Linear elastic - Rule-of-mixtures properties	- Tibial plateau loading	ANSYS/NR	- Stress distribution - Scaffold stress	- Nanoindentation - Compression tests	- Gradient porosity reduces stress peaks
2024, [12]	- Linear elastic bone - Orthotropic lattice	- Distal fixation - 2800 N load	ANSYS/Tetra	- Bone strain - Lattice stress	- Strain-gauge comparison	- 0.8 mm/45° optimal balance
2025, [63]	- Linear elastic - Isotropic materials	- Distal femur fixed - Single-leg stance	ANSYS/NR	- Stress - Displacement	- None	- Pillar lattice lowers stress - Reduced articular displacement

**Table 5:** Overview of experimental studies across mechanical testing, in vitro biology, and in vivo validation (E_mech, E_bio, V) for architected scaffolds and implants in segmental long-bone reconstruction.

Year, Ref [n]	Pathway	Experimental Approach	Test/Assessment	Outputs/Metrics	Duration	Key Outcomes
2013, [1]	E_mech	-Open-porous Ti6Al4V specimens - SLM or machined	- Uniaxial compression (coupon level)	- Effective modulus - Strength - Stress–strain (FE input)	- Single campaign	- Effective properties obtained for FE calibration - Only some designs balanced porosity and stability
2014, [70]	E_mech	- Cadaver femur segmental defect - Empty vs. porous Ti implants (3 stiffness levels)	- Ex vivo axial compression to failure - Plate + implant strain gauges	- Plate strain (3 sites) - Implant strain - Load-sharing metric ($\epsilon_{4}$) - Group statistics	- Single ex vivo test (n = 20; 5/group)	- High variability in load sharing - No significant stiffness-group differences - Fixation setup not sensitive to small stiffness changes
2019, [15]	E_mech	- 3D-printed polymer cubes - Cylindrical scaffold in synthetic femur + plate	- Compression to 500 N - 3D DIC strain mapping	- Effective modulus - Full-field displacement/strain - Strain energy	- Single campaign	- Homogenized model matched global stiffness and strain fields - Practical nondestructive construct-level characterization
2019, [5]	E_mech	- PLA/β-TCP/HA scaffolds - Spherical pores vs. gyroid vs. TO - Multiple porosities	- Compression pre-/post-degradation	- Modulus - Strength - Stress–strain change vs. time - Microstructure observations	- Multi-timepoint (weeks)	- Gyroid and TO retained higher stiffness/strength - Microstructure mitigated degradation-related strength loss
2019, [69]	E_mech	- FDM ABS scaffolds - Layer blocks + CT-based femur segment - Honeycomb interior - 4 raster patterns	- Quasi-static compression - Up to 10% height reduction	- Porosity (2 methods) - Strength (1.76–9.34 MPa) - Modulus (52–212 MPa) - Process–property links	- Single monotonic test	- High porosity achievable (∼83%) - Raster/air gap tune modulus and strength
2019, [2]	E_bio + C	- Human induced membranes - Masquelet stage-2 - Patient-specific Ti cages	- Histology (Masson trichrome) - IHC (vascular/osteogenic markers) - qRT-PCR vs. fascia	- Membrane morphology - IHC staining - Angiogenic/osteogenic gene expression	- Mean 100 d interval (83–119 d) n = 5	- Induced membranes highly vascular - Upregulated angiogenic/osteogenic signals - Supports biological role around Ti cages
2020, [9]	E_mech + V	- TPMS bioceramic implants - Rat femur defect (3 mm) - GP vs. GPRC	- Compression to collapse - In vivo + X-ray/μCT + histology	- Collapse strength/stiffness - Fracture incidence - Bone volume/area inside implant - Bone spatial distribution	- Up to 8 weeks (4–6 wk interim)	- GP: higher porosity, frequent fracture - GPRC: improved integrity - Similar bone amount, different distribution
2021, [14]	E_mech	- 3D-printed porous Ti/Ti-Mg - Region-specific designs	- Quasi-static compression	- Modulus - Stress–strain - FE comparison	- Single campaign	- Experimental modulus close to FE (∼10–15%) - Supports stiffness-matching approach
2021, [6]	E_mech + V	- Ti6Al4V ELI gyroid-sheet scaffold - Rat critical femur defect	- Ex vivo torsion - In vivo μCT + histology	- Torsional stiffness - Torque to failure - BV/TV - Interface histology	- 12 weeks	- Restored substantial torsional stiffness - Strong bone ingrowth - BMP-2 group highest recovery
2021, [4]	E_mech + V	- Patient-specific porous Ti implant - Sheep femur critical defect	- Ex vivo three-point bending - In vivo μCT + histology	- Bending stiffness - Failure load - BV/TV - Interface fusion	- Up to 6 months	- Stable fixation - Extensive bone ingrowth - No implant fracture; stiffness near intact limb
2022, [11]	E_mech	- Composite femur analogues - Patient-specific distal femur implants	- Quasi-static + cyclic loading - Strain gauges - Design comparisons	- Cortical strain - Construct stiffness - Cyclic performance - FE agreement	- Single campaign	- Strain patterns matched FE - Optimized lattice improved physiological strain vs. solid designs
2023, [8]	E_mech + E_bio	- Ti ATS blocks (NP/SP/UP) - With/without acid etching (ST) - Single + assembled stacks - Cells: pre-osteoblasts/hMSCs	- Compression (vertical/lateral) - Cyclic assembled test (150 N, 0.05 Hz) - μCT/SEM/roughness/ protein - Live/dead, migration - ARS + RT-qPCR	- Stiffness/strength - Cyclic durability - Imbibition - Surface metrics - Viability/migration - Osteogenic markers (ALP, COL1, RUNX2, OCN)	- In vitro up to 14 d	- Adequate strength and durability - ST-UP best bio-response - Assembled ATS enabled continuous migration
2023, [17]	E_mech	- CF-PEEK femur surrogate (FDM) - CT-derived geometry	- ISO 7206-4 compression - Quasi-static test	- Load–displacement - Total deformation - Apparent stiffness - FE comparison	- Single campaign	- Test results aligned with FE - CF-PEEK suitable femur surrogate for plate testing
2023, [10]	V	- HA bioceramic scaffold - 15 mm ovine metatarsal - Instrumented circular fixator	- In vivo force monitoring - Gait analysis - CT-based stiffness estimation	- GRF - Fixator/internal forces - Callus stiffness (Kc) - Stiffness terms (Kb,p/Kb,d) - TV, CSA, BMD	- 70–90 d (8 limbs)	- Rapid load-sharing recovery - Stiffness increased with healing - Mechanical monitoring predicts regeneration trajectory
2023, [13]	V	- Ti–19Nb–14Zr lattices - 900/1200 μm unit cells - 3 side-wall designs - Sheep tibia + metatarsal	- 12 weeks in vivo - Ex vivo XCT + ML segmentation	- BII distance - BIC fraction - Bone ingrowth depth/volume - Site comparison	- 12 weeks	- Side-closed designs: strong integration - BII < 10 μm; BIC up to ∼95% - Smaller cells + closure improved ingrowth
2024, [16]	E_mech	- Ti6Al4V ELI porous scaffolds - Radial gradient designs	- Nanoindentation - Compression - Pre-/post-stress relief	- Modulus - Hardness - Strain energy - Compression response	- Single campaign	- Stress relief tuned stiffness and energy absorption - Lower modulus with sufficient strength
2024, [12]	E_mech + E_bio + V	- OLS vs. NLS Ti distal femur implants - Biomech on 3D-printed femur + plate - In vitro MG-63 on discs - In vivo pig implants (solid vs. OLS)	- Biomech: 2800 N + strain gauges - In vitro: MTT at 24/48/72 h - In vivo: CT (2/4/8/ 12 wk) + μCT (9 μm)	- Bone strain vs. design - FE vs. measured strain (14–17%) - Cell viability (OD) - Peri-implant density/area	- In vitro 72 h - In vivo 12 wk	- OLS produced favorable strain range - Good MG-63 compatibility - Higher peri-implant bone formation vs. solid

ALP—alkaline phosphatase; ATS—assemblable titanium scaffold; ARS—alizarin red S; BMP/TGF-beta—bone morphogenetic protein/transforming growth factor-beta signaling; BV/TV—bone volume fraction; COL1—collagen type I; DIC—digital image correlation; GP—*β*-glycerophosphate; GPRC—gyroid-with-ring bioceramic; hMSCs—human mesenchymal stem cells; OCN—osteocalcin; RUNX2—runt-related transcription factor 2; ST—surface-treated (acid-etched); UTM—universal testing machine.

**Table 6:** Translational and clinical overviews (C-flagged studies) on patient-specific and lattice-based reconstructions of large bone defects.

Year, Ref [n]	Indication and Defect Site	Defect Size/Type	Implant/Reconstructive Strategy	N	Follow-Up	Outcomes and Complications (Key Points)
2019, [2]	Post-traumatic femoral segmental bone loss; distal diaphyseal/meta-diaphyseal femur	Critical-sized segmental defects after Grade 3B open fractures or debridement of infected nonunions; multi-centimeter bone loss (up to ≈15 cm)	Two-stage Masquelet protocol: initial PMMA spacer and induced membrane, then patient-specific 3D-printed Ti cage filled with bone graft, fixed with intramedullary nail or lateral locked plate	5	12–33 months (mean 22 months)	- Union achieved in all cases - No deep infection or implant failure - Good limb salvage and function
2021, [4]	Large segmental defects of the spine, pelvis, and femur after tumor resection or trauma	Multi-centimeter load-bearing defects	Individualized porous Ti implants based on implant–bone interface fusion	3	∼12–24 months	- Stable radiographic fusion - No loosening or fracture - No major complications reported

## Data Availability

Some or all of the data and models that support the findings of this study are available from the corresponding author upon request.

## Supplementary Materials

The following supporting information can be downloaded at https://www.mdpi.com/article/10.3390/biomimetics11020145/s1: Table S1: Evaluative Benchmarking of CT-Based Personalization Pipelines; Table S2: Credibility-related reporting of FE and numerical simulation; Table S3: Synthesis of key challenges/limitations and actionable future directions for PSLIs in femoral and tibial reconstruction; Table S4. Concrete methodological options to address fatigue and time-dependent performance gaps.

## Abbreviations

The following abbreviations are used in this manuscript:

ABS	Acrylonitrile butadiene styrene
ALP	Alkaline phosphatase
AM	Additive manufacturing
AO	Arbeitsgemeinschaft für Osteosynthesefragen
ARS	Alizarin Red S
ATS	Assemblable titanium scaffold
BC	Boundary conditions
BIC	Bone–implant contact
BII	Bone–implant interface (distance)
BMD	Bone mineral density
BMP-2	Bone morphogenetic protein 2
BSE-SEM	Backscattered-electron scanning electron microscopy
BV/TV	Bone volume/total volume
C	Clinical evidence (clinical study)
CAD	Computer-aided design
CAE	Computer-aided engineering
CF-PEEK	Carbon-fiber-reinforced poly(ether ether ketone)
COL1	Type I collagen
CPP	Calcium phosphate phase/powder
CT	Computed tomography
DEXA	Dual-energy X-ray absorptiometry
DIC	Digital image correlation
DICOM	Digital Imaging and Communications in Medicine
DIW	Direct ink writing
DMLS	Direct metal laser sintering
DoD	Drop-on-Demand
DOE	Design of Experiments
E_bio	In vitro biological experiments
E_mech	In vitro mechanical experiments
EBM	Electron beam melting
EDM	Electrical discharge machining
ET	Exposure time
FDM	Fused deposition modeling
FE	Finite element
FEM	Finite element method/model
FGM	Functionally graded material
FGL	Functionally graded lattice
FP	Feature parameter
FTIR	Fourier-transform infrared spectroscopy
GPRC	G protein-coupled receptor
GRF	Ground reaction force
HA	Hydroxyapatite
HD	Hatch distance
HIP	Hot isostatic pressing
HU	Hounsfield unit
IHC	Immunohistochemistry
IL-6	Interleukin-6
IM	Intramedullary
LPBF	Laser powder bed fusion
LT	Layer thickness
ML	Machine learning
mPCL–TCP	Medical-grade poly(ϵ-caprolactone)–tricalcium phosphate
MRI	Magnetic resonance imaging
NIH	National Institutes of Health
NLS	Non-lattice structure
NP	Nonporous (ATS unit)
OCN	Osteocalcin
OLS	Optimal lattice structure
PACS	Picture archiving and communication system
PCA	Porous-coated anatomic (knee system)
PCL	Poly(ϵ-caprolactone)
PEEK	Poly(ether ether ketone)
PLA	Polylactic acid
PMMA	Poly(methyl methacrylate)
PolyJet	Photopolymer material jetting process (PolyJet)
PSLIs	Patient-specific lattice implants
RANKL	Receptor activator of NF-κ B ligand
RIA	Reamer–Irrigator–Aspirator
ROI	Region of interest
RT	Room temperature
RT-qPCR	Reverse-transcription quantitative polymerase chain reaction
RUNX2	Runt-related transcription factor 2
RVE	Representative Volume Element
S	Simulation (numerical/FEM)
SA/VR	Surface area-to-volume ratio
SGBR	Scaffold-guided bone regeneration
SLA	Stereolithography
SLM	Selective laser melting
SP	Semiporous (ATS unit)
SEBM	Selective electron beam melting
TCP	Tricalcium phosphate
TGF-β1	Transforming growth factor beta 1
THA	Total hip arthroplasty
Ti6Al4V	Titanium alloy with 6% Al and 4% V
Ti6Al4V ELI	Extra-low interstitial Ti6Al4V
TPMS	Triply periodic minimal surface
TKA	Total knee arthroplasty
TV	Total volume
QA	Quality Assurance
UTM	Universal testing machine
V	In vivo animal experiments
VEGFA	Vascular endothelial growth factor A
XCT	X-ray computed tomography
XRD	X-ray diffraction
YAG	Yttrium aluminum garnet
ZTM14N	Ti–19Nb–14Zr titanium alloy
μCT	Micro-computed tomography
