# Supplementary material for: Patient-Specific Lattice Implants for Segmental Femoral and Tibial Reconstruction (Part 2): CT-Based Personalization, Design Workflows and Validation—A Review
Source: Biomimetics (Basel). 2026 Feb 13;11(2):145. doi: 10.3390/biomimetics11020145 (PMC12938023; doi:10.3390/biomimetics11020145)
Supplement: Supplementary file 1 [file biomimetics-11-00145-s001.zip › biomimetics-4062042-supplementary.pdf]

Supplementary

Supplementary Material: Benchmarking Framework for CT-Based Personalization

Supplementary Note S1: The Three-Pillar Minimum Reporting Set (MRS)

1.1 Overview and Rationale

As demonstrated in the main text, many CT-based personalization pipelines are presented as end-to-end, yet key reporting items remain inconsistently documented. This lack of transparency prevents readers from accurately judging the robustness, reproducibility, and clinical readiness of these workflows. To move beyond a descriptive catalog of literature, we introduce a Minimum Reporting Set (MRS) underpinned by a three-pillar benchmarking framework.

This framework is grounded in recurring limiting factors in patient-specific modeling: segmentation reliability, CT attenuation–based material mapping, and sensitivity to modeling inputs (geometry and boundary conditions), as emphasized in previous methodological syntheses.

1.2 Scoring Methodology

The framework evaluates three independent dimensions of a CT-to-implant workflow. Each pillar is composed of concrete reporting items (four items for Pillars A and C; three to four for Pillar B). Items are scored based on the following scale:

- **0:** Not reported.
- **1:** Partially reported or mentioned without sufficient detail.
- **2:** Clearly and reproducibly reported.

Pillar scores are calculated as the unweighted sum of item scores. Importantly, items are only scored when explicitly documented in the manuscript; otherwise, they are conservatively assigned a score of zero.

1.3 Pillar Definitions

Pillar A: Imaging Fidelity and Calibration (Robustness)

This pillar evaluates the quality and robustness of imaging inputs used to initiate CT-based implant workflows. It considers whether acquisition parameters are reported with sufficient detail to support reproducibility and downstream modeling, including scanner settings, slice thickness or voxel size, reconstruction strategy, and artifact handling. When CT data are used to inform material assignment, this pillar also evaluates whether Hounsfield Unit–based calibration or density mapping is described and justified, as well as whether contralateral mirroring is appropriately motivated.

Across the reviewed studies, imaging fidelity is unevenly reported. Several workflows rely on CT data for patient-specific geometry but provide minimal information on acquisition settings, limiting reproducibility and interpretability. More mature workflows report voxel resolution explicitly and describe threshold-based reconstruction or HU-driven material mapping, which supports more reliable mechanical interpretation. The scoring summarized in Table S1 reflects this variability, with higher Pillar A scores associated with explicit reporting of CT sampling intervals and calibration strategies, and lower scores assigned when CT is mentioned only as a geometric input without technical detail.

Pillar B: Segmentation and Model-Generation Transparency (Reproducibility)

This pillar addresses the transparency of the transformation from raw imaging data to a geometric model suitable for design, simulation, and manufacturing. Evaluation criteria include disclosure of segmentation approaches (e.g., thresholding, region-based methods, or manual editing), description of mesh processing or smoothing steps, specification of export formats, and traceability of the software environment used.

The reviewed workflows show moderate improvement in this area compared to imaging fidelity. Several studies describe DICOM-to-CAD pipelines with explicit segmentation and STL export, supporting partial reproducibility. However, segmentation thresholds, manual interventions, and mesh repair steps are often incompletely documented, and software versioning is inconsistently reported. As reflected in Table S1, Pillar B scores tend to increase when workflows include clearly defined regions of interest or reproducible segmentation logic, and decrease when segmentation is treated as a black box.

Pillar C: Workflow Operability and Quality Assurance (Clinical Scalability)

The third pillar evaluates whether a workflow extends beyond a research demonstration toward clinical operability. Criteria include feasibility of turnaround time, accessibility of the software and manufacturing toolchain, explicit consideration of manufacturability constraints for porous architectures, and the presence of any quality assurance or verification loop.

Most reviewed workflows score lowest in this dimension. While some studies describe virtual planning steps, contralateral mirroring, or basic manufacturability considerations, explicit quality assurance measures and standardized clinical handoff procedures are uncommon. A small subset of studies report geometry verification or micro-CT–based inspection, supporting benchmarking of QA practices rather than full clinical pipelines. As summarized in Table S1, higher Pillar C scores correspond to workflows that explicitly consider production constraints and verification steps, whereas lower scores reflect research-oriented pipelines without defined QA or clinical integration.

1.4 Tier Classification

Based on cumulative scores across the three pillars, workflows are classified into three hierarchical tiers:

- **Tier 1 (Prototype / Low Reproducibility):**

  Imaging, segmentation, and modeling steps are incompletely reported; workflows rely heavily on manual intervention; calibration and quality assurance are absent.

- **Tier 2 (Reproducible Research Pipeline):**

  Key acquisition, segmentation, and export steps are documented at a minimum level; workflows include at least one reproducible element such as defined regions of interest, HU-based mapping, or partial validation, but lack full clinical operability.

- **Tier 3 (Clinically Scalable Pipeline):**

  Workflows combine standardized imaging documentation, transparent and reproducible segmentation, explicit manufacturability constraints, a quality assurance loop, and a clear handoff toward clinical use.

This tiered classification, summarized in Table S1, makes explicit how differences in reporting quality and workflow completeness affect the readiness of CT-based implant pipelines for reproducible research and clinical translation.

Table S1: Evaluative Benchmarking of CT-Based Personalization Pipelines

The following table operationalizes the MRS by scoring representative pipelines across Pillars A, B, and C, assigning Tier labels, and providing justifications based on explicit manuscript documentation.

Ref [n], Year	Pillar A (0–8)	Pillar B (0–8)	Pillar C (0–8)	Tier	One-line Justification
[68], 2018	1	1	2	Tier 1–2	Patient-specific CT-based reconstruction described; acquisition parameters and segmentation rules are minimally documented.
[2], 2019	2	0	4	Tier 2	CT-based virtual planning and contralateral mirroring support operability; acquisition and segmentation parameters are underreported.
[3], 2020	0	0	0	Tier 1	CT mentioned for defect definition, but acquisition settings, segmentation, export formats, and QA are not documented.
[14], 2021	2	2	0	Tier 1–2	Element-wise HU-based material assignment reported; CT acquisition parameters and segmentation thresholds largely omitted.
[17], 2023	1	4	1	Tier 1–2	DICOM-to-Mimics workflow with STL/CAD transfer improves transparency; calibration and automation reporting remain limited.
[10], 2023	5	3	3	Tier 2	Voxel size, threshold-based segmentation, and phantom-based HU-to-BMD calibration reported; clinical handoff partially described.
[13], 2023	3	4	2	Benchmark	Best-practice example for XCT acquisition and ML-based segmentation for QA, though not a full personalization pipeline.
[66], 2023	0	0	2	Benchmark	Micro-CT parameters reported for geometry verification, supporting QA criteria for microarchitecture fidelity.
[63], 2025	4	5	0	Tier 2	CT sampling interval and HU-to-density-to-modulus mapping explicitly reported; segmentation and STL export described; lacks clinical handoff and QA.

Table S2: Credibility-related reporting of finite element (FE) and numerical simulation

This table summarizes the credibility-related reporting of finite element (FE) and numerical simulation studies across the included studies. Fields capture analysis type, interface/contact definitions, bone material modeling, nonlinearities, validation level, and an overall fidelity tier (Tier 1–3) used for study comparison.

Ref [n], Year	Analysis type	Interfaces (type + μ)	Bone modeling	Nonlinearities (geom/material)	Validation level	Fidelity tier
[1], 2013	FE of femur–scaffold–plate	Bone–scaffold slip prevented (relative displacement	Inhomogeneous bone from CT HU mapping	Material nonlinearity for porous Ti (elastic–	Quantitative validation (FE deflection ~95%	3

Ref [n], Year	Analysis type	Interfaces (type + μ)	Bone modeling	Nonlinearities (geom/material)	Validation level	Fidelity tier
	construct	inhibited effectively tied/bonded); μ not used	(element-wise properties)	plastic behavior implemented)	agreement with experiment; scaffold properties from mechanical tests)	
[65], 2015	Nonlinear static (large-displacement)	Multiple contacts: screw–bone μ=0.23, nail–cancellous μ=0.10; also tied constraints used	Homogeneous cortical/cancellous (no HU mapping used)	Geom nonlinearity ON; contact nonlinearity; material linear elastic	No new experiment (comparisons to literature patterns)	2
[68], 2018	Static FE (design-stage construct evaluation)	NR (contact types not reported in FE description)	Cortical bone + soft callus region (healing region modeled)	Material linear elastic (reported); Geom: NR	None (FE used to guide design; no error metric)	2
[67], 2018	Static structural FE	NR (assembly described, but no explicit contact/tie/friction definition stated)	CT-based geometry; homogeneous properties (HU mapping not stated)	Linear elastic; Geom: NR	None (parametric concept study)	1
[5], 2019	Simulation present (Abaqus mentioned), FE setup underreported	NR	Not a bone–implant construct (microstructure/scaffold focus); bone modeling NA	NR	NR (no clear FE-test metric reported in described Abaqus sections)	1
[15], 2019	Quasi-static FE (detailed vs homogenized scaffold)	Tie constraints (threads not modeled, tied to holes); screw–plate μ=0.5; scaffold–plate μ=0.3	Composite femur surrogate with assigned cortical/cancellous properties (not HU-mapped)	Contact nonlinearity; material linear elastic for polymer scaffold bulk + homogenized orthotropic equivalent	Quantitative (compression tests + 3D DIC; ~9% stiffness error for homogenized vs detailed)	3
[3], 2020	Nonlinear FE across healing stages	Stage 1: frictional contact μ=0.2; Stages 2–3: bonded implant–bone	Patient-specific geometry; HU mapping	Contact nonlinearity; healing stage material reassignment; Geom: NR	None stated (numerical case study)	2
[4], 2021	Static 3-point bending FE	NR in main text (materials/details appear in Supplement Table S1)	CT-based sheep femur segment geometry; HU mapping not stated in main text	NR	Quantitative: stiffness/displacement correlation FE vs experiment ($R^2 \approx 0.96$)	3
[14], 2021	Static structural FE	Bonded bone–implant (μ not used)	CT-based HU-ρ-E mapping (heterogeneous element-wise)	Material: linear elastic; Geom: NR	Quantitative (strain-gauge + compression test; mesh sensitivity)	3
[7], 2022	Static FE (unit cell + femur construct	Bonded femur–scaffold interface stated; μ not used	Homogeneous cortical/cancellous (no HU mapping reported)	Material nonlinearity present (elasto-plastic curve used for scaffold);	None stated	2

Ref [n], Year	Analysis type	Interfaces (type + μ)	Bone modeling	Nonlinearities (geom/material)	Validation level	Fidelity tier
	comparisons)			Geom: NR		
[11], 2022	Static structural FE	Implant–femur No separation; screw interfaces Bond (μ not used)	Homogeneous cortical/cancellous (no HU mapping reported)	Contact-type constraints; material linear elastic	Quantitative in vitro biomechanical tests with strain gauges compared to FE	3
[62], 2023	Quasi-static FE	Bone–screw frictional contact μ=0.4; scaffold–bone and screw–plate binding constraints; plate–bone contact constraint	Homogeneous cortical/cancellous (no HU mapping reported)	Contact nonlinearity; material linear elastic	None (explicitly states no experiments)	2
[8], 2023	Linear static FE (scaffold-only)	Rigid scaffold–scaffold interface between assembled units; μ not used/NR	Bone modeling NA	Linear elastic	Qualitative consistency with mechanical tests (no explicit error metric)	1–2
[17], 2023	Static structural FE	Interfaces NA (single femur model)	Homogeneous/isotropic femur (no HU mapping reported)	Material linear elastic; Geom: NR	None stated in methods/results	1–2
[12], 2024	Quasi-static FE (gait/single-leg-stance based loading)	Frictional contact between bone–screw, plate–screw and lattice–bone (μ not reported)	Cortical + cancellous; lattice modeled via RVE-derived orthotropic properties	Contact nonlinearity; material plasticity/damage NR; Geom: NR	Quantitative strain-gauge validation (~14–17% difference)	3
[16], 2024	Static structural FE	NR (no contact definition stated in FE section)	CT-based tibia geometry; HU mapping not stated (likely homogeneous regions)	Linear elastic; Geom: NR	Property-level support (nanoindentation/compression), no direct FE output validation stated	2
[63], 2025	Static structural FE	Femur–plate bonded; femur–screws bonded (μ not used)	CT-based HU→E mapping stated	Linear elastic; Geom: NR	None (patient-specific numerical study)	2

Table S3: Synthesis of key challenges/limitations and actionable future directions for patient-specific lattice implants (PSLIs) in femoral and tibial reconstruction

Theme	Current gap / limitation	Why it matters (mechanistic / translational impact)	Practical future direction(s)	Minimum reporting items (benchmarking)
Clinical workflow and regulation	CT-based planning and lattice design often assume	Delays and inconsistency hinder routine adoption and	Semi-automatic segmentation/design/validation pipelines	Time-to-design, software/tools, decision

Theme	Current gap / limitation	Why it matters (mechanistic / translational impact)	Practical future direction(s)	Minimum reporting items (benchmarking)
	ideal timelines; workflows are variable across centers; fragmented regulatory expectations; limited registries.	comparability; unclear documentation and follow-up weakens evidence.	integrated into hospital systems; prospective registries; standardized follow-up pathways.	points, regulatory pathway, follow-up schedule, outcome definitions.
Imaging and segmentation	Resolution limits for thin cortices and trabecular detail; operator-dependent segmentation; limited reporting of observer variability; bone quality and soft tissue captured only coarsely.	Personalization may be geometrically correct but biologically incomplete; uncertain defect-edge quality affects fixation and interface behavior.	Quantify segmentation variability; add density/directionality/necrosis/previous-implant maps; define consensus CT/μCT metrics for peri-implant vs intraporous bone.	Scanner settings, voxel size, segmentation method, inter/intra-observer variability, ROI definitions, thresholds/phantoms, BV/TV/BIC/BII metrics.
Design space and standardization	Many lattice choices with partial parameter reporting; ad hoc design variable selection; limited cross-site comparisons; lack of reference designs.	Hard to build consensus on pore/relative density/gradient strategies across defect types; slows translation and reproducibility.	Reporting standards for lattice and defect description; DOE/surrogate optimization; libraries of reference femur/tibia designs; automated constraint checking (resection margins, fixation footprint, safety windows).	Unit cell type/size/orientation, gradients (what/where/why), surface treatment, defect length/location, fixation method, constraints used.
Simulation fidelity and uncertainty	Many models use linear elasticity, simplified interfaces, static loads, limited physiologic boundary conditions; weak validation; limited uncertainty quantification.	Static "safe stress" may overestimate long-term stability; bonded contacts may hide loosening; patient-load variability is ignored.	Multiaxial loading, nonlinear/contact modeling where relevant; multiscale frameworks; probabilistic/sensitivity analyses; tighter coupling to measurable bench/clinical readouts (strain gauges, motion, serial imaging).	Load cases/BCs, interface/contact definition, material model, mesh convergence, validation target (strain/stiffness), uncertainty sources and ranges.
Experimental and biological evidence	Mechanical tests often simplified and not fully segment-representative; in vitro focuses on early markers; limited long-term vascular/immune response; animal models may not reflect human segmental complexity.	Evidence may demonstrate feasibility but not durability or advantage over standard of care; long-term interface behavior remains uncertain.	Standardized construct-level tests (combined bending/torsion/compression); longer-term biology endpoints; comparative studies vs plates/nails/cages/transport; define "minimum intraporous bone" thresholds for safety.	Test configuration, load mode, failure definition, endpoints, timepoints, species/model, comparison group, graft/biologics use.
Manufacturing robustness and quality control	AM defects, anisotropy, surface roughness, batch	Fatigue life and stiffness can shift markedly with small strut	In-process monitoring plus acceptance criteria; nondestructive evaluation	Machine, parameter set, post-processing, surface

Theme	Current gap / limitation	Why it matters (mechanistic / translational impact)	Practical future direction(s)	Minimum reporting items (benchmarking)
	variability; limited process monitoring reporting; internal lattice defects hard to detect.	deviations; undetected defects threaten safety and reproducibility.	(µCT/ultrasound/thermography where feasible); qualification and re-qualification protocols for printer/powder/post-processing.	state, defect inspection method, acceptance thresholds, batch traceability.
Data, AI and decision support	Data are sparse, heterogeneous, not reusable; limited shared datasets linking imaging-design-simulation-testing-follow-up.	Slows benchmarking and prevents decision-support tools that could guide design/fixation choices and risk estimation.	Shared databases/registries linking CT/design/FE/bench/in vivo/clinical; common schemas; risk models for failure/loosening/nonunion under rehab scenarios.	Data schema, feature definitions, missingness, outcome measures, sharing policy, center/site metadata.
Clinical adoption and outcomes	Limited cost-effectiveness evidence; heterogeneous outcomes and follow-up; single-center experiences dominate.	Adoption depends on reproducibility, cost, and clear indications/contraindications with long-term results.	Standardized outcome sets (union, time to weight bearing, revisions, PROMs); cost-benefit analyses; long-term follow-up especially for tumor/infection cases.	Outcome definitions, follow-up duration, rehab protocol, complications, revision criteria, PROMs, cost elements.

Table S4. Concrete methodological options to address fatigue and time-dependent performance gaps.

Gap	Proposed methodology	What it yields (measurable outputs)	Key reporting items
Fatigue absent	Coupon-level fatigue plus construct-level cyclic loading	S–N and/or strain–life envelope; stiffness retention; run-out rate; construct stiffness drop	R ratio, frequency, cycles, environment, failure criterion, n, surface/post-processing state
Crack initiation unclear	In situ µCT fatigue testing (or staged cyclic plus repeat µCT)	3D crack initiation/growth in struts; defect evolution vs stiffness drop; pore connectivity changes	Imaging interval, voxel size, ROI, segmentation thresholds
FE lacks time-dependence	Multiscale fatigue FE (global construct plus local submodel/RVE)	Hotspot stress/strain; predicted fatigue life; damage accumulation; architecture sensitivity	BC realism, mesh/convergence, calibration dataset, validation metric
Interface durability underreported	Cyclic micromotion measurement plus longitudinal imaging	Micromotion/slip trends; loosening indicators; BV/TV/BMD/BIC trajectories linked to mechanics	Micromotion method (DIC/DVC/sensors), timepoints, ROI definitions, thresholds, follow-up duration
